# Propagation of Photoinduced Electric Field Changes Through Phytochrome and their Impact on Conformational Transitions

**DOI:** 10.1002/cphc.202500595

**Published:** 2025-09-21

**Authors:** Mariafrancesca La Greca, Anh Duc Nguyen, Anastasia Kraskov, Norbert Michael, Luisa Sauthof, Manal Ebrahim, Sagie Katz, Johannes von Sass, Oan Tu Hoang, Nediljko Budisa, Patrick Scheerer, Ramona Schlesinger, Maria Andrea Mroginski, Peter Hildebrandt

**Affiliations:** ^1^ Experimental Physics: Genetic Biophysics Freie Universität Berlin Arnimallee 14 D‐14195 Berlin Germany; ^2^ Institut für Chemie, Sekr. PC7 Technische Universität Berlin Straße des 17. Juni 135 D‐10623 Berlin Germany; ^3^ Institut für Chemie, Sekr. PC14 Technische Universität Berlin Straße des 17. Juni 135 D‐10623 Berlin Germany; ^4^ Institute of Medical Physics and Biophysics Group Structural Biology of Cellular Signaling Charité − Universitätsmedizin Berlin, corporate member of Freie Universität Berlin and Humboldt‐Universität zu Berlin Charitéplatz 1 D‐10117 Berlin Germany; ^5^ Department of Chemistry University of Manitoba 44 Dysart Rd Winnipeg Manitoba R3T 2N2 Canada

**Keywords:** electric fields, molecular modelings, phytochromes, Stark effect, vibrational spectroscopies

## Abstract

In phytochromes, photoisomerization of the chromophore and subsequent structural relaxations lead to the functionally essential secondary structure transition of the tongue, a phytochrome‐specific protein segment. The coupling mechanism between chromophore and protein structural changes is yet not understood, but electric field changes are discussed to play an important role. In this work, electric field changes in the chromophore binding pocket (CBP) are confirmed to propagate over long distances through the protein and alter the electric field in the tongue region. An experimental‐theoretical approach to analyze local electric fields using Stark reporters has been further developed. These are nitrile groups introduced site‐specifically into the protein via noncanonical amino acids. The functional integrity of the variants is checked by crystallography and various spectroscopies. For the first time, functionally intact variants with substitutions in the tongue are generated. Based on frequency shifts and relative intensities of the nitrile stretching modes, hydrogen‐bonding and noncovalent electric field contributions are separated. The field changes originating in the CBP are transduced to the tongue along a pathway via Phe192. Given a proper direction of the net electric field vector in the tongue region, the magnitude of the field may be sufficient to destabilize the tongue structure.

## Graphical abstract

Photoconversion of phytochrome causes structural alterations in the chromophore binding pocket, associated with local electric field changes, which are analyzed by experimental and theoretical methods. The field changes propagate to the distant tongue region of the protein. Magnitude of the field changes may be an important factor contributing to the functionally essential secondary structure transition of the tongue.

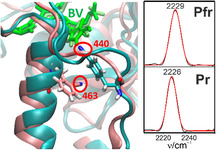

## 1 Introduction

**Figure 1:**
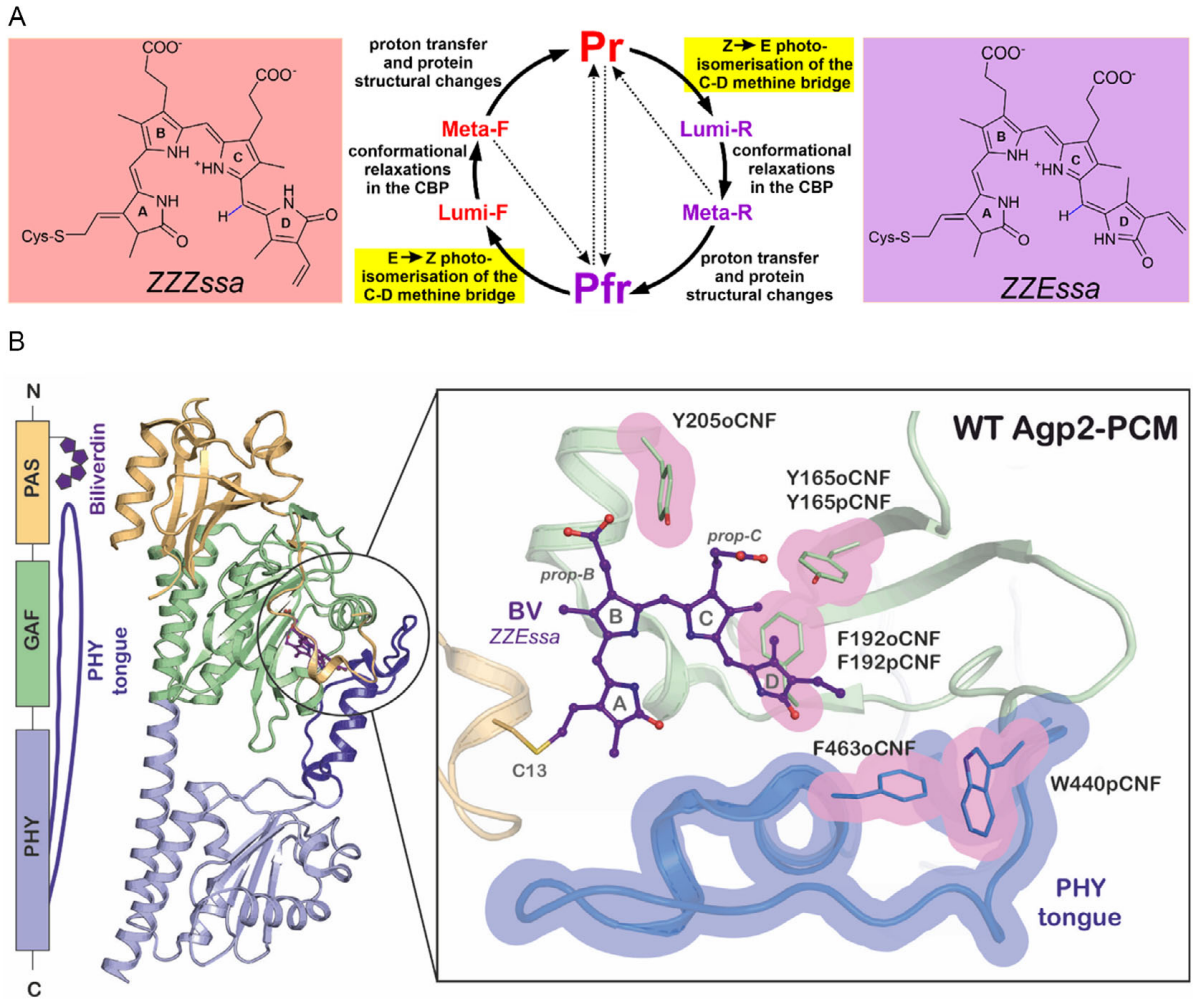
A) Simplified reaction scheme of the photoconversion of phytochromes, including a brief description of the key events of the individual reaction steps. The configuration of the BV chromophore is ZZZssa and ZZEssa in the states marked in red and violet, respectively. The photochemical steps are highlighted in yellow whereas all other reactions are thermally activated. In the dark, Pr is the stable state in prototypical phytochromes and Pfr in bathy phytochromes, as in Agp2 studied in this work. B) Left, domain composition and structure of Pfr of WT Agp2‐PCM;^[^ ^32^ ^]^ right, close‐up view showing the CBP and the tongue region showing those amino acids that were substituted by pCNF and oCNF (highlighted in pink frames). The protein backbone, chromophore, and selected residues of WT Agp2‐PCM (PDB‐ID 6G1Y) are shown as cartoon, sticks/balls, and sticks, respectively.

Phytochromes constitute a class of biological photoreceptors which harbor a linear methine‐bridged tetrapyrrole as a chromophore.^[^ ^1^, ^2^, ^3^, ^4^, ^5^ ^]^ They are ubiquitous in plants where they use light to trigger physiological processes related to plant growth and development.^[^ ^1^, ^3^ ^]^ In addition, representatives of the phytochrome family were also discovered in bacteria and fungi.^[^ ^6^, ^7^, ^8^ ^]^ All phytochromes share essential structural and mechanistic properties.^[^ ^5^, ^9^ ^]^ The minimum functional entities are the photosensor (photosensory core module (PCM)) and an output module which is frequently a histidine kinase.^[^ ^2^, ^10^ ^]^ The PCM includes the chromophore which upon light absorption undergoes a double bond isomerization of the methine bridge double bond between rings C and D (C—D methine bridge; **Figure** **1**).^[^ ^5^ ^]^ This event is followed by conformational relaxations in the chromophore binding pocket (CBP) that eventually leads to the secondary structure conversion of the tongue, a phytochrome‐specific protein segment.^[^ ^11^ ^]^ The structural change of the tongue triggers further protein structural changes in the PCM, which in the last step are transmitted to the output module to (de)activate the catalytic site. Phytochromes are photoswitches interconverting between the parent states Pr and Pfr, in which the chromophore adopts a ZZZssa and ZZEssa configuration, respectively.^[^ ^5^ ^]^ The transition between Pr and Pfr may also proceed via thermal routes (Figure 1A) and the ratio between the respective rate constants determines either Pr or Pfr as the thermodynamically stable (dark) states, defining the group of prototypical and bathy phytochromes, respectively.^[^ ^12^ ^]^

In our previous studies, we intensively used the bathy phytochrome Agp2 from *Agrobacterium fabrum* as a model system,^[^ ^13^ ^]^ providing valuable insight into structure and reaction mechanism of phytochromes in general. Agp2 carries a biliverdin (BV) chromophore, which upon light excitation of the dark state Pfr photoisomerizes from ZZEssa to ZZZssa and subsequently runs via a sequence of thermal decays to Pr. In this reaction sequence, the Meta‐F intermediate plays a key role since it constitutes a branching point with two possible reaction pathways.^[^ ^14^, ^15^, ^16^ ^]^ One is a shortcut that directly returns to the initial state Pfr without affecting the tongue structure, whereas the other one proceeds to the final photoproduct Pr via the structural transition of the tongue from α‐helix/coil to β‐hairpin/sheet. Only the latter reaction route is functionally relevant since it couples the structural changes of the PCM and the catalytic output module. A conditio sine qua non to open this route is the proton transfer from the ring C propionic side chain (propC) of BV to His278, as revealed by a comprehensive investigation of variants with site‐specific substitutions in the CBP.^[^ ^17^ ^]^ The proton transfer represents a charge separation generating a high local electric field. Therefore, these findings,^[^ ^17^ ^]^ together with further experimental results,^[^ ^18^ ^]^ prompted the hypothesis that changes of the local electric fields, originating in the CBP, are an important driving force for the secondary structure transition of the tongue.

To check this hypothesis, we have exploited the vibrational Stark effect (VSE) that describes the perturbation of vibrational energy levels and transitions by an external electric field.^[^ ^19^, ^20^, ^21^, ^22^, ^23^ ^]^ The VSE is preferentially observed for highly localized modes such as the stretching of the nitrile group. In fact, this mode has been widely used for studying the VSE since it offers the additional advantage that the stretching frequency is in a region free of any other fundamental modes and thus easy to detect. However, the use of nitriles as VSE probes is associated with two major challenges.

First, the nitrile stretching frequency does not only respond to noncovalent electric fields but also to hydrogen‐bonding (HB) interactions, albeit in opposite directions.^[^ ^21^, ^24^, ^25^ ^]^ Thus, it imposes substantial difficulties on the evaluation of electric field effects in a HB environment. This obstacle can be overcome by taking into account the second observable of the nitrile stretching mode, the IR band intensity.^[^ ^21^ ^]^ This quantity allows for determining the total electric field sensed by the nitrile, regardless of its origin in noncovalent or HB interactions. Using both observables, approaches have been developed to separate the two contributions to the electric field.^[^ ^21^, ^26^ ^]^

Second, the nitrile probe must be introduced at appropriate positions in the protein. An elegant approach is the site‐specific incorporation of nitrile groups via noncanonical amino acids, such as *para*‐ or *ortho*‐cyano‐phenylalanine (pCNF, oCNF).^[^ ^23^, ^26^, ^27^, ^28^, ^29^ ^]^ In our previous studies we have focused on Tyr165 and Phe192 in the CBP of Agp2 (Figure 1B).^[^ ^26^, ^28^ ^]^ These residues play important roles in coupling structural changes in the CBP and the tongue either as a part of the proton transfer chain (Tyr165) or a conformational switch (Phe192). Substituting these residues by pCNF and oCNF we could assess the electric field sensed by the nitrile groups at two positions with different orientations during the individual steps of the photoconversion. However, the results obtained so far did not allow unambiguous conclusion about the possible effect on the tongue segment.

In this work, we reveal that the electric field changes in the CBP of phytochromes are propagated over long distances through the protein and alter the electric field in the tongue region. To uncover this striking result, we strategically placed VSE probes far from the CBP, at both termini of the tongue (Trp440 and Phe463), pushing the spatial limits of electric field sensing. Additionally, we introduced a probe at Tyr205, a site that modulates the interactions between the B‐ and C‐ring propionic side chains during photoconversion—a critical step preceding propC deprotonation. We then applied our integrated experimental‐theoretical approach, previously established,^[^ ^22^ ^]^ and now refined, to probe these subtle yet far‐reaching electrostatic effects for the first time.

## 2 Results and Discussion

### 2.1 Concept and Methodology

The analytical concept consists of four steps.^[^ ^26^ ^]^ First, structural and functional integrity of the Agp2 variant were carefully examined by various spectroscopic techniques. For each variant a structural model of the Pfr state was generated either by protein X‐ray crystallography or molecular modeling, starting from the wild‐type (WT) Agp2‐PCM structure. Second, for the Pfr state that is used as a reference for the subsequent analysis, the local averaged electric field projected onto the VSE probe and the vibrational transition dipole moment (TDM) of the nitrile stretching mode are calculated by quantum‐mechanical molecular‐mechanical (QMMM) hybrid methods. Third, for all states of the photoconversion, frequencies and intensities of the nitrile stretching relative to the Pfr reference states were measured and extrapolated to room temperature. Forth, the combined use of experimental normalized frequencies and intensities as well as calculated reference TDMs constitute the basis to evaluate the electric fields experienced by the nitrile probe due to HB or noncovalent interactions.

### 2.2 Structural and Functional Integrity of the Variants

The three variants Y205oCNF, W440pCNF, and F463oCNF, are functionally and structurally largely intact according to structural models and spectroscopic criteria, although there are some minor differences compared to WT Agp2‐PCM. They all undergo a photoconversion, show the tongue restructuring coupled to the deprotonation of propC, and display very similar chromophore structures. For Y205oCNF, the crystal structure of Pfr was determined experimentally at a resolution of 1.79 Å (Table S2, Supporting Information). This structure reveals a very high overall agreement, even in the CBP, with a root‐mean‐square deviation (RMSD) of 0.28 Å compared to the WT Agp2 PCM (Figure S1, Supporting Information). However, introducing the oCNF reporter group leads to an in‐place rotation of the Y205oCNF side chain by ≈20° compared to Tyr205 in WT‐Agp2 PCM (Figure S1, Supporting Information). Together with the lack of the hydroxyl head group in oCNF, this alteration leads to a slight restructuring of the HB network around the BV chromophore in the Pfr crystal structure. On the one hand, a new water molecule is visible in the oCNF region of CBP, on the other hand, there is a slight shift in the head group of propC and a different rotamer orientation of Ser260 (Figure S1, Supporting Information). Sampling of the conformational space through molecular dynamics (MD) simulations revealed only minor structural fluctuations of oCNF and its surrounding environment. This stability is reflected in the low RMSD of 1.617 ± 0.16 Å relative to the crystal structure, indicating that the simulated conformations remain closely aligned with the experimentally observed arrangement (Figure S2, Supporting Information).

**Figure 2:**
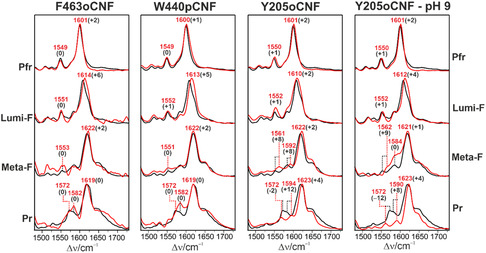
RR spectra in the C=C stretching region of the three variants (red) compared with the WT Agp2‐PCM (black). Red labels denote the peak maxima in the variant spectra whereas black numbers in parentheses indicate the frequency difference with respect to the corresponding peaks of the WT Agp2‐PCM. The spectra were measured with 1064 nm excitation at 90 K. Photoinduced states were obtained by irradiating (750 nm) the sample at 140 K (Lumi‐F), 240 K (Meta‐F) and 293 K (Pr) prior to cooling to 90 K. Residual contributions of the unphotolyzed state Pfr were subtracted on the basis of the characteristic Pfr marker bands.

For the other two variants, we generated structural models for the Pfr state by in silico substitutions of Try440 and Phe463 by pCNF and oCNF, respectively. Subsequent MD simulations and geometry optimization yielded models with differences only at the substitution sites (Figure S3 and S4, Supporting Information). In the F463oCNF models, the nitrile reporter group induces a rotation of residue 463. This rearrangement is driven by strong HB interactions between the reporter group and the backbone amide of Tyr191. In the W440pCNF models, substitution of the bulky tryptophan in the relatively flexible loop region with pCNF leads to significant conformational changes in the surrounding environment. Presumably driven by HB interactions, Lys446 flips toward the nitrile group, accompanied by a rearrangement of Pro444. RMSDs of heavy atoms in the environment of the Stark probe, relative to the crystal structure, are 1.782 ± 0.141 Å for the F463oCNF and 2.170 ± 0.367 Å for the W440pCNF model. QM/MM calculations also predict greater mobility of the pCNF unit compared to oCNF, consistent with spectroscopic data (vide infra). Since Tyr205 directly interacts with the propionic side chains, initially with propB and propC in Pfr and subsequently in Meta‐F solely with propB, one may readily rationalize the effects of the substitution on the chromophore, which is visible through small changes in the Y205oCNF Pfr crystal structure. These effects include Q‐band shifts in the UV–vis absorption spectra by 6 nm (Figure S4, Supporting Information) and differences in the resonance Raman (RR) spectra that were noted for a few bands but do not exceed 4 cm^−1^, with the exception of Lumi‐F (**Figure** **2**; Figure S5 and S6, Supporting Information).

Here, a quite substantial frequency downshift of the hydrogen out‐of‐plane (HOOP) mode of the C—D methine bridge is observed. In contrast to Y205oCNF, the substitution sites of W440pCNF and F463oCNF are at the tongue segment and thus not in contact with the chromophore (Figure 1B). Hence, it is surprising that also these variants reveal spectral changes related to the chromophore, both in the UV–vis absorption and the RR spectra (Figure 2; Figure S4, S7, and S8, Supporting Information). The changes are qualitative and quantitatively similar as for Y205oCNF or the variants with substitutions at Tyr165 and Phe192.^[^ ^26^, ^28^ ^]^ Interestingly, substitution of Trp440 by pCNF or Phe seems to lower the quantum yield of the Pfr → Lumi‐F transition since prolonged irradiation is required for a substantial extent of photoconversion. In the RR spectra of Pfr and Meta‐F only relatively small effects were found for all variants, while again Lumi‐F displays distinctly larger frequency shifts for the HOOP and the C=C stretching modes of the C—D methine bridge. The frequencies of the HOOP and C—D stretching modes are known to be highly sensitive markers for the single and double bond torsions of the C—D methine bridge.^[^ ^30^ ^]^ In view of such notable effects also in W440pCNF and F463oCNF, we thus conclude that the distorted C—D methine bridge in Lumi‐F delicately depends on subtle structural or electrostatic perturbations that originate from even remote centers in the protein. A similar explanation may account for the enol‐keto equilibrium in Pr,^[^ ^31^ ^]^ which differs from WT Agp2‐PCM in all three variants (Figure 2, Figure S5–S8, Supporting Information). As judged from the enol marker bands at ≈1250 and 1585 cm^−1^, the enol portion in Pr decreases in the order F463oCNF > W440pCNF > WT > Y205oCNF. In addition, the tautomeric equilibrium of Y205oCNF shows a peculiar behavior since increasing pH to 9.0 causes an increase of the enol portion only in Meta‐F but not in Pr.

**Figure 3:**
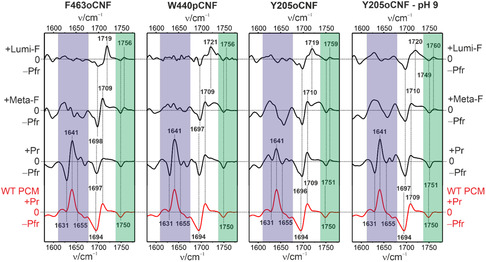
IR difference spectra of the variants (black) compared with the WT Agp2‐PCM (red). The blue‐ and green‐shaded regions mark the amide I bands and the C=O stretching of the protonated propionic side chain.

The increasing contribution of the enol tautomer is also reflected by the decreasing intensity of the ring D C=O stretching (≈1700 cm^−1^) in the IR difference spectra although a quantitative evaluation is more difficult due to overlapping bands of opposite sign (**Figure** **3**). The most important spectral regions in the IR difference spectra, however, are the amide I modes (1620–1660 cm^−1^) and the C=O stretching of the protonated propC (1750–1760 cm^−1^). In all variants, the propC group remains protonated and the secondary structure of the protein unchanged from Pfr to Meta‐F. In W440pCNF and F463oCNF—as in WT Agp2‐PCM—the transition to Pr is associated with the deprotonation of propC, as indicated by the loss of the positive signal at ≈1760 cm^−1^.^[^ ^31^ ^]^ Concomitantly, we observe distinct positive signal at ≈1640 cm^−1^ accompanied by two weaker negative bands at ≈1630 and 1655 cm^−1^, reflecting the conversion of the tongue from the α‐helix/coil (negative signals) to the β‐hairpin/sheet structure (positive signals). In Y205oCNF, this coupled process is not completed at pH 7.8. It requires an increase of the pH to 9.0 to achieve the same signal pattern as in the other variants and in WT Agp2‐PCM.

### 2.3 Analysis of the Nitrile Stretching Modes

**Figure 4:**
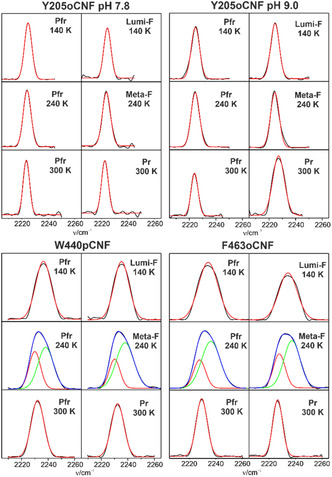
IR spectra in the nitrile stretching region of the Y205oCNF at pH 7.8 and pH 9.0, W440pCNF, and F463oCNF variants. The spectra of the photoproducts were obtained after subtracting the residual Pfr state contribution from the measured spectrum and subsequent band fitting. The experimental curves are given by the black lines whereas red and green traces represent the fitted Gaussian functions. In case of a two‐Gaussian fit, the blue line refers to the sum of the individual Gaussians. The spectra of the photoproduct states, obtained at the indicated temperatures, were normalized to the conjugate spectra of the Pfr state.

IR spectra in the nitrile stretching region were measured for the parent state and the photoinduced cryo‐trapped states such that at each temperature a reference spectrum of Pfr was obtained (**Figure** **4**). This procedure offers three advantages. First, the spectrum of the trapped species could be “purified” by appropriate subtraction of the residual unphotolysed Pfr contribution. The subtraction factor was typically between 10% and 50% and thus similar as in the RR experiments where comparable sample concentrations were used. The choice of the factor was further guided by the attempt to obtain symmetric bandshapes of the resultant difference spectrum that could be simulated by a Gaussian fit. This subtraction procedure was also applied to the variants studied in our previous work (Supporting Information, section 4.1; Figure S9, Table S3 and S4, Supporting Information).^[^ ^26^, ^28^ ^]^ Second, for each variant, intensities of each state were related to the intensity of the Pfr reference state. Third, relative intensities and frequencies were normalized to room temperature assuming the same temperature dependence of the band parameters for the photoinduced states Lumi‐F, Meta‐F, and Pr as for Pfr (Table S3 and S4, Supporting Information).^[^ ^26^ ^]^ In most cases, the bandshapes allowed for satisfactory fits using a single Gaussian. In this sense, the band widths reflect conformational broadening due to a Gaussian distribution, consistent with the MD simulations. Nevertheless, the band widths were relatively small. Together with the previously studied variants,^[^ ^26^, ^28^ ^]^ we found a clear tendency for the band widths at room temperature with 5.5 and 8.7 cm^−1^ for the oCNF and pCNF nitrile modes, respectively (Table S6, Supporting Information). Most likely, the intrinsic mobility of the nitrile substituent is lower in the *ortho* position as shown by MD simulations, thereby accounting for a decreased conformational broadening. Only for W440pCNF and F463oCNF as well as Y205oCNF at pH 9 a further broadening by ≈2 cm^−1^ compared to the other pCNF and oCNF variants was observed. In a few cases, two‐Gaussian functions were required for an acceptable fit, indicating two distinct population pools differing in few conformational coordinates or HB interactions.

### 2.4 Calculations of the Pfr Reference States

All three variants fulfill the requirement that each variant i) undergoes the functionally relevant proton‐coupled secondary structure transition and ii) shows a Pfr state which is structurally largely unchanged compared to the WT Agp2‐PCM. Thus, we can choose the structural models of the Pfr state as a reference to calculate the averaged electric fields projected onto the nitrile bonds of the Stark label ($E_{F,Pfr,tot}$) and the TDM of the nitrile stretching mode ($\left|{\vec{m}}_{Pfr,tot}\right|$). We have taken the average of 25 QMMM calculations of snapshots from MD simulations (Supporting Information, Section S4.3). Interestingly, the calculated average electric fields of Pfr are much larger for the “tongue” variants W440pCNF and F463oCNF than that of the CBP variant Y205oCNF (Table S7, Supporting Information). The electric field and the TDM are related to each other according to

(1) $$\left|{\vec{m}}_{i}\right|=E_{F,i}\cdot A+\left|{\vec{m}}_{0}\right|$$

where $\left|{\vec{m}}_{0}\right|$ is the TDM in the absence of a field and *A* is a proportionality factor. Plotting the data sets of the individual snapshot calculations according to Equation (1) (*i* = Pfr), $\left|{\vec{m}}_{0}\right|$ and *A* can be determined (Table S8, Figure S10, Supporting Information).

### 2.5 Evaluation of the Electric Fields

Determination of the electric fields follows the procedure introduced previously, including some modifications as described in the Supporting Information, Section S4. The relative normalized intensities (vide supra) of the entire band envelopes of each state $I_{i,tot}$ and the calculated TDMs for Pfr $\left|{\vec{m}}_{Pfr,tot}\right|$ were used to evaluate the total TDM for all states *i* of each variant $\left|{\vec{m}}_{i,tot}\right|$ according to

(2) $$\left|{\vec{m}}_{i,tot}\right|=\sqrt{\frac{I_{i,tot}}{I_{Pfr,tot}}}\left|{\vec{m}}_{fr,tot}\right|$$

where $I_{Pfr,tot}$ is the reference intensity of Pfr. Then, the corresponding electric fields $E_{F,i,tot}$ were evaluated via

(3) $$E_{F,i,tot}=\frac{\left|{\vec{m}}_{i,tot}\right|-\left|{\vec{m}}_{0}\right|}{A}$$

An analogous procedure was applied to states including two substates *j*. Here we evaluated not only the individual $\left|{\vec{m}}_{i,j}\right|$ and $E_{F,i,j}$, but also the mole fractions $x_{j}$ of the substates. All values for $\left|{\vec{m}}_{i,j}\right|$ and $x_{j}$ are listed in Table S5 (Supporting Information). Note that averaged electric field quantities that are calculated by QMMM or derived from experimental data are amounts of the corresponding vectors. They carry a negative sign when they contribute to energy lowering as it is usually the case. For the sake of simplicity, increasing and decreasing electric field strength therefore refer to more negative or less negative quantities, respectively. The electric fields of all states $E_{F,i,tot}$ and their substates $E_{F,i,j}$ were then separated into contributions from noncovalent (subscript “non”) and HB (subscript “HB”) interactions using an approximation developed previously.^[^ ^26^ ^]^ This approach is described in detail in the Supporting Information (Section S4.4). It assumes that both contributions combine additively to the frequency shift $\Delta \nu_{i}$ (in ${\text{cm}}^{\text{‐1}}$) and is based on the linear VSE for noncovalent interactions (Equation 4) and an analogous expression for H‐bonded nitriles (Equation 5)^[^ ^25^ ^]^

(4) $$\Delta \nu_{i,non}=|\vec{\Delta \mu }|E_{F,i,non}$$

(5) $$E_{F,i,non}=\frac{\Delta \nu_{i}-k\cdot E_{F,i,tot}}{|\vec{\Delta \mu }|-k}$$

with the Stark tuning rate $|\vec{\Delta \mu }|=0.37{\left(\text{MV}/\text{cm}\right)}^{-1}{\text{cm}}^{‐1}$ and $k=0.2\;{\left(\text{MV}/\text{cm}\right)}^{-1}{\text{cm}}^{‐1}$. The empirical parameter *k* is the counterpart to $\left|\vec{\Delta \mu }\right|$ for H‐bonded systems,^[^ ^25^ ^]^ and its numerical value was adjusted in our previous study.^[^ ^25^ ^]^ Note that in this work we have redetermined the value for $\left|\vec{\Delta \mu }\right|$ as described in the Supporting Information, Section S4.2. The corresponding HB‐related electric field is then given by

(6) $$E_{F,i,HB}=E_{F,i,tot}-E_{F,i,non}$$

**Figure 5:**
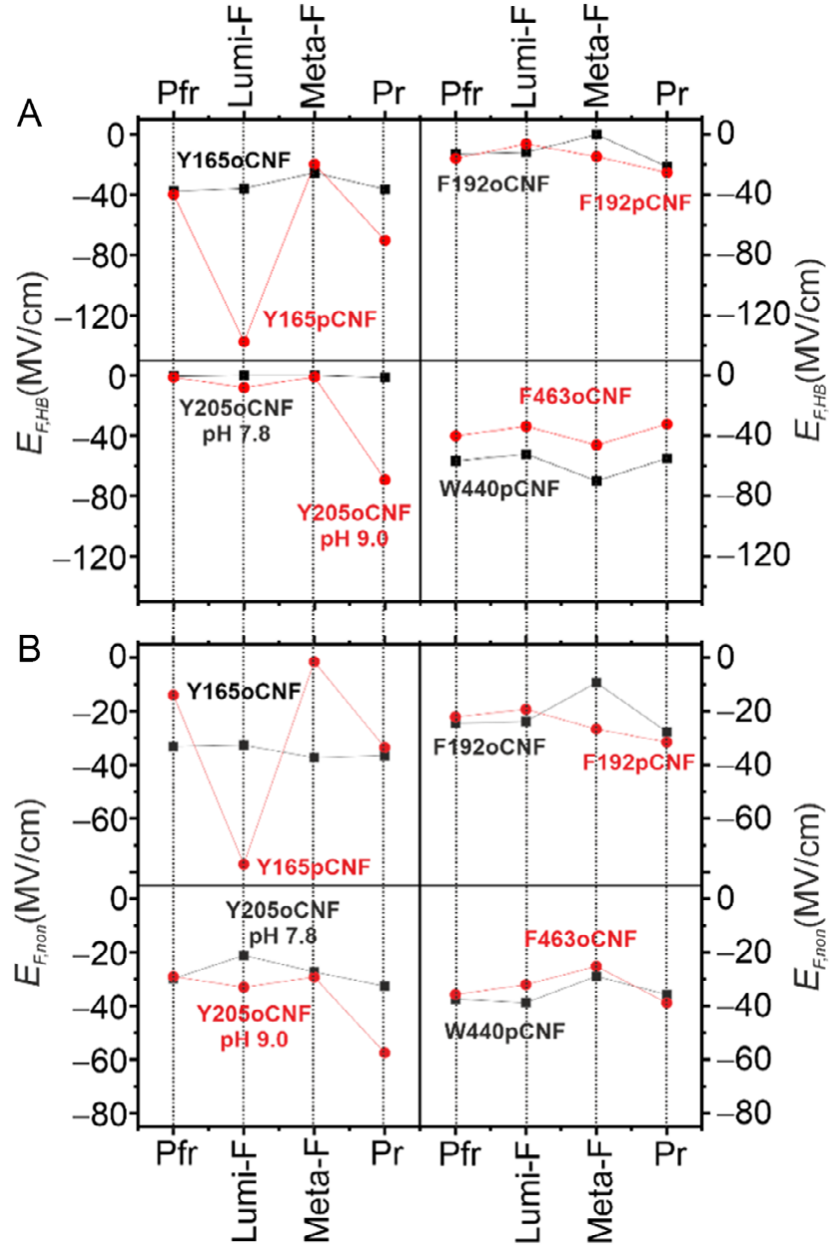
Representation of the A) HB related and B) noncovalent electric fields in the various states of the Pfr → Pr photoconversion of Agp2. The individual data is listed in Table 1, including variants already previously reported,^[^ ^26^, ^28^ ^]^ but reanalyzed together with the variants presented in this work.

**Figure 6:**
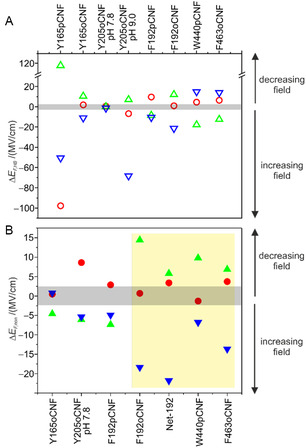
Differences in the electric fields for the individual reaction steps indicating “Lumi‐F−Pfr” in red, “Meta‐F−Lumi‐F” in green, and “Pr−Meta‐F” in blue. A) HB‐related field differences (open symbols). B) Noncovalent electric field differences (full symbols). The gray‐shaded regions in (A) and (B) mark differences which are zero or close to zero, taking into account the approximate errors. In (B), the data for Y165pCNF and Y205oCNF (pH 9.0) was not considered as discussed in the text. Net‐192 refers to the net electric field determined for the residue 192 according to Equation (7) and (8). The pale‐yellow area highlights variants with common patterns of noncovalent electric field changes. The individual data is taken from Table 1 and 2, including for variants already previously reported,^[^ ^26^, ^28^ ^]^ but reanalyzed together with the variants presented in this work.

All values for $E_{F,i,tot}$, $E_{F,i,j}$ and the respective noncovalent and HB contributions are given in **Table** **1** and displayed in **Figure** **5** and **6**.

**Table 1:** Noncovalent and HB‐related electric fields (in MV cm^−1^ ; subscripts "non" and "HB") and nitrile stretching frequencies (in cm^−1^) of the various states and substates of all variants.

	$E_{F,tot}$ a)	P1					P2					$E_{F,tot,non}$	$E_{F,tot,HB}$
		*ν*_1_	$x_{1}$	$E_{F,1}$	$E_{F,1,non}$	$E_{F,1,HB}$	*ν*_2_	$x_{2}$	$E_{F,2}$	$E_{F,2,non}$	$E_{F,2,HB}$		
Y165oCNF													
Pfr	−70.79	2229.8	1.0	−70.79	−33.08	−37.71	–	–	–	–	–	−33.08	−37.71
Lumi‐F	−68.45	2229.6	1.0	−68.45	−32.61	−35.84	–	–	–	–	–	−32.61	−35.84
Meta‐F	−62.59	2225.8	1.0	−62.59	−37.22	−25.37	–	–	–	–	–	−37.22	−25.37
Pr	−72.82	2228.3	1.0	−72.82	−36.43	−36.38	–	–	–	–	–	−36.43	−36.38
F192oCNF													
Pfr	−37.36	2226.6	0.72	−37.08	−26.87	−10.21	2231.8	0.28	−38.07	−18.10	−19.98	−24.41	−12.95
Lumi‐F	−35.71	2226.5	0.69	−39.38	−27.85	−11.53	2231.7	0.31	−27.55	−14.58	−12.97	−23.74	−11.98
Meta‐F	−9.31	2227.0	1.0	−9.31	−9.31 (−16.42)b)	0.0 (7.12)b)	–	–	–	–	–	−9.31	0.0
Pr	−49.03	2226.2	0.55	−48.32	−31.52	−16.80	2231.4	0.45	−49.90	−22.95	−26.95	−27.66	−21.37
Y165pCNF													
Pfr	−53.70	2237.3	1.00	−53.70	−13.93	−39.77	–	–	–	–	–	−13.93	−39.77
Lumi‐F	−214.50	2233.5	1.00	−214.50	−77.02	−137.48	–	–	–	–	–	−77.02	−137.48
Meta‐F	−21.13	2237.9	1.00	−21.13	−1.45	−19.68	–	–	–	–	–	−1.45	−19.68
Pr^i^	−103.66	2232.0	0.63	−103.28	−40.62	−62.66	2243.3	0.37	−104.31	−21.16	−83.15	−33.42	−70.24
F192pCNF													
Pfr	−38.06	2229.5	1.0	−38.06	−22.13	−15.93	–	–	–	–	–	−22.13	−15.93
Lumi‐F	−25.56	2225.4	0.4	−26.91	−25.41	−1.50	2230.8	0.6	−24.65	−15.14	−9.51	−19.25	−6.31
Meta‐F	−41.35	2227.6	1	−41.35	−26.62	−14.74	–	–	–	–	–	−26.62	−14.74
Pr	−56.79	2227.9	1	−56.79	−31.50	−25.28	–	–	–	–	–	−31.51	−25.28
Y205oCNF													
Pfr	−30.33	2223.6	1	−30.33	−29.77	−0.57	–	–	–	–	–	−29.76	−0.57
Lumi‐F	−21.16	2223.3	1	−21.16	−21.16 (−27.07)b)	0 (5.91)b)	–	–	–	–	–	−21.16	0
Meta‐F	−27.28	2223.7	1	−27.28	−27.28 (−28.52)b)	0 (1.24)b)	–	–	–	–	–	−27.28	0
Pr	−33.97	2222.7	1	−33.97	−32.62	−1.35	–	–	–	–	–	−32.62	−1.35
Y205oCNF pH9													
Pfr	−30.33	2224.0	1	−30.33	−29.06	−1.27	–	–	–	–	–	−29.06	−1.27
Lumi‐F	−41.14	2223.9	1	−41.14	−33.09	−8.11	–	–	–	–	–	−33.09	−8.11
Meta‐F	−30.27	2223.9	1	−30.27	−29.22	−1.05	–	–	–	–	–	−29.22	−1.05
Pr	−126.77	2227.1	1	−126.77	−57.46	−69.31						−57.46	−69.31
W440pCNF													
Pfr	−94.10	2232.0	1	−94.10	−37.40	−56.70	–	–	–	–	–	−37.40	−56.69
Lumi‐F	−90.86	2230.6	1	−90.86	−38.72	−52.14	–	–	–	–	–	−38.72	−52.13
Meta‐F	−98.91	2232.1	0.28	−99.58	−39.15	−60.43	2240.0	0.72	−98.64	−24.96	−73.68	−28.94	−69.97
Pr	−90.83	2232.3	1	−90.83	−35.73	−55.10	–	–	–	–	–	−35.73	−55.10
F463oCNF													
Pfr	−75.89	2229.3	1	−75.89	−35.75	−40.14	–	–	–	–	–	−35.75	−40.14
Lumi‐F	−65.80	2229.4	1	−65.80	−32.03	−33.76	–	–	–	–	–	−32.03	−33.76
Meta‐F	−72.37	2229.3	0.46	−54.53	−28.26	−26.27	2238.8	0.54	−85.83	−22.57	−63.26	−25.19	−46.25
Pr	−71.18	2226.6	1	−71.18	−38.84	−32.34	–	–	–	–	–	−38.84	−32.35

a) $E_{F,tot}$, Total electric field of the peaks 1 and 2 derived from the intensities (sum of the fields $E_{F,1}$ and $E_{F,2}$).

b) In few cases of electric fields of largely noncovalent character. Equation (3) $E_{F,non,i}=\frac{\Delta \nu -k\cdot E_{F,i}}{|\vec{\Delta \mu }|-k}$ lead to positive values for the HB‐related fields (in parentheses), which were then set to zero. The corresponding value for the noncovalent field was then set equal to the total field of the respective peak.

The nature of $E_{F,HB}$ and $E_{F,non}$ is qualitatively different. $E_{F,HB}$ results from direct interactions of the nitrile group with HB‐donors and thus mirrors the HB changes during the photoinduced reaction sequence. This can clearly be seen for variants at position 165. In Pfr of the WT protein, Tyr165 together with propC, Tyr205, and His278 forms a HB network that is perturbed upon rotation of the C—D methine bridge (Figure 1). Subsequently, it is involved in the proton transfer relay from propC to His278. In Y165pCNF but not in Y165oCNF, the nitrile group takes over the function of the hydroxyl group of Tyr as reflected by the large changes of $E_{F,HB}$. Accordingly, Y165pCNF is not an innocent or noninvasive reporter. In each reaction step, $E_{F,HB}$ variations of Y165pCNF are much larger than those of the other variants, which are mainly in a more hydrophobic environment (Figure 6). It is therefore tempting to assume that except for Y165pCNF, the smaller $E_{F,HB}$ changes reflect variable interactions with water molecules rather than durable and strong HB with amino acid residues (Figure 5). One exception is Y205oCNF where the nitrile substituent in *ortho* position in Y205oCNF cannot replace the hydroxyl group of Tyr and its HB interactions, first with propB and propC (Pfr) and later solely with propB (Meta‐F).^[^ ^32^, ^33^ ^]^ However, upon raising the pH to 9.0, which is required for a complete propC deprotonation, only weak HB interactions are detected in the first and second reaction step but a drastic increase of $E_{F,HB}$ is observed for the Meta‐F to Pr transition. Most likely, the pH increase affects the protein structure with consequences for the kind of HB interactions. Accordingly, both Y205oCNF at pH 9.0 and Y165pCNF, due to its invasive character, were not considered for the further analysis.

**Figure 7:**
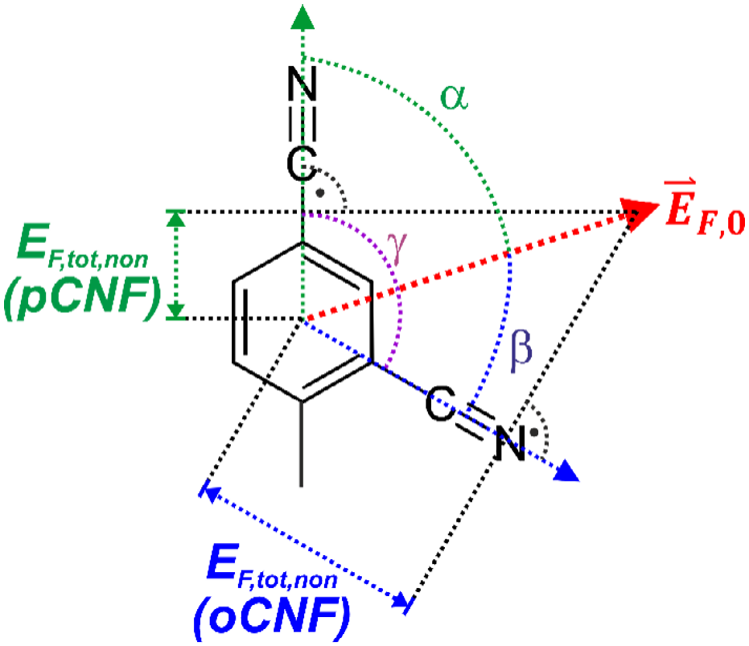
Definition of the net electric field ${\vec{E}}_{F,0}$ at residue 192. The quantities $E_{F,tot,on}$ (pCNF) and $E_{F,tot,non}$ (oCNF) are the noncovalent electric fields determined from the Stark labels of F192pCNF and F192oCNF, respectively. The angles between ${\vec{E}}_{F,0}$ and the vector along the nitrile bonds of F192pCNF and F192oCNF are given by α and β, respectively. The angle between the nitriles in para and ortho position is given by *γ* = 120°.

In contrast to the through‐bond contacts associated with $E_{F,HB}$, $E_{F,non}$ reflects the effect of forces acting through space on the nitrile bond. Charges and dipoles in the vicinity of a Stark probe generate an electric field ${\vec{E}}_{F,0}$. The values for $E_{F,non}$, as listed in Table 1, represent the projection of ${\vec{E}}_{F,0}$ onto the nitrile bond but magnitude and orientation of ${\vec{E}}_{F,0}$ remain unknown. These two quantities, however, can be determined for two Stark probes at the same position but different orientations in the protein. This is in principle the case for the variant pairs with pCNF or oCNF at positions 192 and 165. Unlike to the conjugate pair at position 165, which is either an “invasive” or a field‐insensitive probe, Phe192 is in a largely hydrophobic pocket and the respective variants F192pCNF and F192oCNF are involved only in very weak HB interactions (Table 1). Moreover, in Pfr position and orientation of these residues relative to the chromophore are essentially the same as for the unsubstituted Phe, as indicated by the structural models. The far‐reaching similarity of the structures further supports the view that also the local electrostatics is not significantly altered by replacing Phe through the *para*‐ or *ortho*‐substituted Phe. Based on this assumption, we determined the net electric field vector ${\vec{E}}_{F,0}$ in this part of the protein. Accordingly, ${\vec{E}}_{F,0}$ may form approximately the same angle α with respect to the main (rotational) C_2_ axis of these residues intersecting the ring through the atoms in para position, including the nitrile bond in pCNF (**Figure** **7**). For the corresponding *ortho*‐substituted residue, the angle with respect to ${\vec{E}}_{F,0}$ is (120 − *α*) degrees. Using the electric fields $E_{F,non}$ evaluated for F192pCNF and F192oCNF (Table 1), corresponding to the projection of ${\vec{E}}_{F,0}$ onto the respective nitrile bonds, one obtains with $E_{F,0}$ as the amount of ${\vec{E}}_{F,0}$

(7) $$E_{F,Pfr,non}\left(pCNF\right)=cos\alpha \cdot E_{F,0}$$

and

(8) $$E_{F,Pfr,non}(oCNF)=\text{cos}(120-\alpha )\cdot E_{F,0}$$

Combining Equation (7) and (8) allows determining the angle *α* to 61.3°. The magnitude of the net electric field $E_{F,0}$ is then evaluated to −46.5 MV cm^−1^ (**Table** **2**).

**Table 2:** Net electric field $E_{F,0}$, calculated according Equation (6)and (7), noncovalent electric field $E_{F,tot,non}$ , taken from Table 1, and the angles between the corresponding vectors for the various states of the Phe192 variants.

		Pfr	Lumi‐F	Meta‐F	Pr
F192pCNF	$E_{F,tot,non}$/(MV cm^−1^)	−22.13	−19.25	−26.62	−31.51
	α/degree	61.6	63.5	44.5	57.8
	$E_{F,0}$/(MV cm^−1^)	−46.53	−43.14	−37.32	−59.13
F192oCNF	$E_{F,tot,non}$/(MV cm^−1^)	−24.41	−23.74	−9.31	−27.66
	β/degree	58.4	56.5	75.5	62.2
	$E_{F,0}$/(MV cm^−1^)	−46.53	−43.14	−37.32	−59.13

### 2.6 Electric Field Changes in the CBP

The first reaction step from Pfr to Lumi‐F is initiated by the photoisomerization of the BV chromophore, followed by structural adjustments of the amino acids the HB network in the immediate environment of the chromophore.^[^ ^33^, ^34^ ^]^ Except for $E_{F,non}$ of Y205oCNF, the concomitant changes of noncovalent electric fields are small for all variants and for Y165oCNF even zero. This is consistent with the calculation of the net electric field vector in Lumi‐F at position 192 with *α* = 63.5° and $E_{F,0}=-43.1\;{\text{MV cm}}^{–1}$, similar as in Pfr (Table 2).

In the second reaction step (Lumi‐F → Meta‐F), the noncovalent field changes are more pronounced at all three positions in the CBP. They can be rationalized in terms of the structural changes in Meta‐F that are required for proton transfer from propC (Tyr205 and Tyr165),^[^ ^31^ ^]^ and the conformational switch (Phe192) to induce restructuring of the tongue,^[^ ^32^ ^]^ that both occur in the final reaction step.^[^ ^17^ ^]^ In Meta‐F of Phe192 and the respective substituted variants, reorientation is reflected by the rotation the phenyl ring with respect to the net electric field vector ${\vec{E}}_{F,0}$ by ≈20° (Table 2).

The last reaction step from Meta‐F to Pr is associated with the proton transfer from the neutral propC to the neutral His278, corresponding to a charge separation. Hence, we expect that the labels at three positions in the CBP sense particularly large changes of noncovalent electric fields. This in fact the case for the reporter groups at position 192 which do not only indicate an increase of the field in Pr but also a rotation of the net field vector with respect to the phenyl ring by ≈13° compared to Meta‐F (Table 2). Conversely, the changes of the noncovalent field are only moderate for Y205oCNF and even close to zero in Y165oCNF, presumably due to unfavorable orientations with respect to the net electric field vector (Table 1, Figure 6). Interestingly, the HB‐related electric field changes, which are rather small in the two preceding reaction steps, increase in Pr at positions 165 and 192, presumably due to water influx into the CBP (Figure 6).

### 2.7 Electric Field Changes in the Tongue Region

Previous crystallographic and spectroscopic studies did not provide any indication for structural changes of the tongue in the first two reaction steps from Pfr to Meta‐F. This is also in line with the present spectroscopic analyses. Thus, one would expect that electric field changes are restricted to the CBP. Instead, the tongue variants W440pCNF and F463oCNF (Figure S3, Supporting Information) report both HB‐related and noncovalent field changes that are in each reaction step comparable or even larger in magnitude than those of the CBP variants (Table 1, Figure 5 and 6). Changes of the HB interactions of W440pCNF and F463oCNF are presumably due to different water contacts; they are first weakened in Lumi‐F, then strengthened in Meta‐F, and become finally weaker again with the formation of Pr. The latter effect can readily be attributed to an altered solvation of the tongue in the β‐sheet structure. However, HB changes in the first two reaction steps indicate a long‐range structural communication pathway from the epicenter of the photoinduced field changes, the isomerization site, to the tongue region (Table S10, Supporting Information). This conclusion also holds for the noncovalent electric field changes. Such a pathway may be considered as a consecutive series of repositioning (partial) charges, reorientations or dipoles, and subtle displacements of amino acid residues such that the electric field change, generated in the CBP, is eventually transduced to the region of the tongue.

Inspection of the data in Figure 6B demonstrates that the noncovalent field changes of W440pCNF and F463oCNF are not random but display the same pattern as the net electric field at residue 192 (Figure 6B), implying that the field changes at these three residues are “in phase”, that is, a decrease and increase of the field with formation and decay of Meta‐F, respectively. Consequently. we conclude that Phe192 is an important corner point of the field transduction route from the CBP to the tongue. This conclusion is consistent with Phe192´s role in the switch that couples conformational changes of the CBP with the tongue.^[^ ^32^ ^]^ Finally, these observations further suggest that the net field vectors at residues Trp440 and Phe463 point in similar directions as that at Phe192.

We now ask if the photoinduced electric field changes that are propagated to the tongue region are functional for the secondary structure transition. In fact, electric fields as an origin for inducing protein structural changes have been discussed in various studies previously, ranging from model peptides to protein segments and aggregates.^[^ ^35^, ^36^, ^37^, ^38^, ^39^, ^40^, ^41^, ^42^, ^43^ ^]^ In most cases, the underlying idea is that electric fields may destabilize specific HB interactions that are characteristic of a given secondary structure pattern. For α‐helices, these are the HB interactions between the amide C=O group of residues *i* and the amide N—H group of residues *i *+ 4. In a recent systematic study of a model peptide, Ilieva et al. showed that for an appropriate directionality electric fields of ≈25 MV cm^−1^ are sufficient to weaken and eventually disconnect the attractive forces between the conjugate amide bonds.^[^ ^35^ ^]^ A study on bovine insulin as a real protein reports that even weaker electric fields (6–10 MV cm^−1^) destroy the native secondary structure.^[^ ^36^ ^]^ Although the interactions of amino acid side chains that are additional structure‐stabilizing factors may differ from protein to protein, the previous results may define an approximate critical range from 5 to 25 MV cm^−1^ in which external electric fields can affect the structure of protein domains.

In Agp2, the secondary structure transition of the tongue starts with the destabilization of the coil region, presumably at the end of the Meta‐F lifetime,^[^ ^32^ ^]^ and is completed with the formation of Pr.^[^ ^31^ ^]^ Formation of Meta‐F is associated with a decrease of the noncovalent electric field at the nitrile groups of W440pCNF and F463oCNF by 7 to 10 MV cm^−1^. Taking into account that the amount of the corresponding net electric field vector ${\vec{E}}_{F,0}$ is larger than its projection onto the nitrile group, the changes of the electric field strength in the tongue region may weaken the structure of the tongue and thus contribute to its transformation, given a favorable direction of the vector ${\vec{E}}_{F,0}$. In this way, electric field changes may be an important factor for the functional conformational transition in phytochromes.

## 3 Conclusions

Based on a combined experimental and theoretical approach, the local electric fields at seven Stark reporter groups in the CBP and the tongue region were analyzed in terms of HB‐related and noncovalent contributions. Except for the nitrile group of Y165pCNF that exactly adopts the role the hydroxyl function in Tyr165, the $E_{F,HB}$ changes of the other reporter groups reflect different degrees of interactions with water.

Photoinduced electric field changes can propagate over considerable distances through the protein, probably via many consecutive steps of charge and dipole reorientations and displacements. The field transduction seems to run through a pathway with Phe192 as a hinge between the CBP and the tongue region. This electrostatic and conformational coupling does not only account for the electric field changes at the tongue in response to the photoinduced processes in the CBP but also explains the effect of tongue mutations on the electronic and structural properties of the BV chromophore, as revealed by UV–vis and RR spectra.

The noncovalent field changes at the nitrile functions of the tongue variants W440pCNF and F463oCNF, which are monitored upon formation of Meta‐F, are between 7 and 10 MW cm^−1^. Given a favorable direction of the corresponding net electric field vectors ${\vec{E}}_{F,0}$, the magnitude of the field change may be sufficient to induce the structural transformation of the tongue from α‐helix to β‐sheet.

## Supporting Information

The experimental and theoretical methods and supplementary data (Tables S1–S10 and Figures S1–S11) are given in the Supporting Information. The authors have cited additional references in the Supporting Information.^[13,21,27,44–68]^

## Conflict of Interest

The authors declare no conflict of interest.

## Supporting information

Supplementary Material

## Data Availability

The data that support the findings of this study are available from the corresponding author upon reasonable request
