# Supplementary material for: Propagation of Photoinduced Electric Field Changes Through Phytochrome and their Impact on Conformational Transitions
Source: Chemphyschem. 2025 Sep 21;26(22):e202500595. doi: 10.1002/cphc.202500595 (PMC12640670; doi:10.1002/cphc.202500595)
Supplement: Supplementary file 1 — Supplementary Material [file CPHC-26-e202500595-s001.pdf]

**Supporting Information**

# Propagation of photoinduced electric field changes through phytochrome and their impact on conformational transitions

Mariafrancesca La Greca,[a] Anh Duc Nguyen,[b] Anastasia Kraskov,[c] Norbert Michael,[c] Luisa Sauthof,[d] Manal Ebrahim,[d] Sagie Katz,[c] Johannes von Sass,[c] Oanh Tu Hoang,[b] Nediljko Budisa,[e] Patrick Scheerer,[d] Ramona Schlesinger,[a] Maria Andrea Mroginski,[b*] Peter Hildebrandt[c*]

[a] Freie Universität Berlin, Experimental Physics: Genetic Biophysics, Arnimallee 14, D-14195 Berlin, Germany

[b] Technische Universität Berlin, Institut für Chemie, Sekr. C7, Straße des 17. Juni 115, D-10623 Berlin, Germany

[c] Technische Universität Berlin, Institut für Chemie, Sekr. PC14, Straße des 17. Juni 135, D-10623 Berlin, Germany

[d] Charité − Universitätsmedizin Berlin, corporate member of Freie Universität Berlin and Humboldt-Universität zu Berlin, Institute of Medical Physics and Biophysics, Group Structural Biology of Cellular Signaling, Charitéplatz 1, D-10117 Berlin, Germany

[e] Department of Chemistry, University of Manitoba, 144 Dysart Rd, R3T 2N2 Winnipeg, Manitoba, Canada

**Content**

**1. Methods**

**1.1. Protein expression**

**1.2. Spectroscopy**

**1.3. Protein crystallography**

**1.4. Computational methods**

**2. Structural data**

**2.1. Crystal structure of Y205oCNF**

**2.2. Structural models of F463oCNF and W440pCNF**

**3. Spectroscopic data**

**3.1. UV-vis absorption spectroscopy**

**3.2. Resonance Raman spectroscopy**

**3.3. IR Spectroscopy**

**4. Analysis of the nitrile stretching modes**

**4.1. Spectra subtraction**

**4.2. Stark tuning rate**

**4.3. Calculated electric fields and transition dipole moments**

**4.4. Non-covalent and hydrogen-bonding-related fields**

**5. References**

# 1. Methods

## 1.1. Protein expression

### 1.1.1. Agp2-PCM W440pCNF

60 ng/µL of the plasmid pET21bAgp2W440TAG containing a TAG codon at position Trp-440 (Table S1) was cotransformed with 60 ng/µL of the pEVOL-aaRS vector containing two copies of the tRNA/aaRS pair of the archaeal *Metanococcus jannaschii* into *E. coli* strain BL21(DE3). The cells were then transferred to 500 mL flasks containing TB media supplemented with ampicillin (200 µg/mL) and chloramphenicol (34 µg/mL) at 37 °C and 0.2 % arabinose from a 20 % stock solution in Milli-Q water. When the cell culture reached an $OD_{600}$ of 0.5-0.6, 4 mM of p-cyano-phenylalanine was added from a stock solution of 0.2 M in 0.5 N NaOH. When the $OD_{600}$ reached 0.8-1, expression was induced with 1 mM IPTG and incubated overnight at 20°C. The next day, expression was harvested at 10,000xg for 10 min (Avanti J-26 XP , Beckman, Germany). The cell pellet was washed twice with 50 mM Tris pH 7.8, 400 mM NaCl, 5 % glycerol and once with 50 mM Tris pH 7.8, 50 mM NaCl, 5 % glycerol before disruption. After centrifugation at 45,000 xg for 20 minutes (Optima L-80 XP, Beckman, Germany), the supernatant was applied to a Ni-NTA column (Macherey-Nagel GmbH & Co. KG, Germany) using Äkta (Avant 25, GE Healthcare). The His-tagged apoprotein was washed and eluted using 50 mM Tris pH 7.7, 300 mM NaCl, 10 mM imidazole, 2 % glycerol and 50 mM Tris pH 7.7, 400 mM NaCl, 400 mM imidazole, 2 % glycerol. The eluate was concentrated and applied to a size exclusion column (Superdex 200, 10/300 GL, GE Healthcare) using 20 mM HEPES pH 7.5, 150 mM NaCl , and the apoprotein was reconstituted with the 3-molar excess of biliverdin previously dissolved in methanol and the latter buffer. The mixture of apoprotein and biliverdin was incubated overnight at 4°C and run on another size exclusion column (HiLoad™ Superdex 200 16/60 prep grade) to separate the fraction of chromophore-bound protein from the free biliverdin. The concentration was then determined by UV/Vis spectroscopy.

### 1.1.2. Agp2-PCM W440F.

60 ng/µL of the plasmid pET21bAgp2W440F (Table S1) was transformed into the *E. coli* strain BL21(DE3)Codon$^{+}$ RP. The cells were then transferred to 500 ml flasks containing TB media supplemented with ampicillin (200 µg/mL) at 37°C. When the cell culture reached an $OD_{600}$ of 0.8 - 1, expression was induced with 0.5 mM IPTG and incubated overnight at 20°C. All subsequent steps were the same as described above.

### 1.1.3. Agp2-PCM Y205oCNF and Agp2-PCM F463oCNF

Expression and purification of these variants followed the same protocol as described previously.[1]

**Table S1.** Primers used for constructing Agp2-PCM Y205oCNF and Agp2-PCM F463oCNF via QuikChange PCR

| | **Forward primer** | **Reverse primer** |
|---|---|---|
| **Agp2W440F:** | 5´- CGTGCAGAACCTGAAC **TTT**GCCGGCAATCCGG - 3´ | 5´- CCGGATTGCCGGC **AAA**GTTCAGGTTCTGCACG - 3´ |
| **Agp2W440TAG** | 5´- CGTGCAGAACCTGAAC **TAG**GCCGGCAATCCGG - 3´ | 5´- CCGGATTGCCGGC **CTA**GTTCAGGTTCTGCACG - 3´ |

## 1.2. Spectroscopy

### 1.2.1. UV-Vis absorption spectroscopy

UV-vis absorption spectra were measured either using a Varian Cary 50 Bio UV−vis spectrophotometer (Agilent), a Maya 2000 Pro spectrometer (Ocean Optics), or a Shimadzu® UV-2600i. The protein sample was typically diluted to an OD at 750 nm of ∼0.25. The sample was converted to the Pr state by illumination by LED light between 750 and 780 nm for 3 - 5 minutes.

### 1.2.2. Vibrational spectroscopy

For spectroscopic experiments, dark-adapted protein in Tris-buffered solution (pH 7.8) was concentrated to ca. 1 mM for RR and IR difference spectroscopy, and to ca. 10 mM for the IR measurements in the nitrile stretching region. For the latter, the sample was additionally semi-dried to form a 4 µm thick protein film that was sandwiched between two $CaF_2$ plates. Prior to the experiments, the sample was fully converted to the Pfr state by illumination with a 670 nm LED. RR measurements were performed using a Bruker Fourier-transform Raman spectrometer RFS 100/S or a Bruker Fourier-transform MultiRAM spectrometer with a Ramanscope III. Both spectrometers were equipped with a Nd-YAG cw laser for 1064 nm excitation (line width 1 $cm^{-1}$) (Bruker, Karlsruhe, Germany) with 1064 nm excitation (Nd-YAG cw laser, line width 1 $cm^{-1}$), equipped with a nitrogen-cooled cryostat from Resultec (Linkam). All spectra of the samples in frozen solution were recorded at ca. 90 K with a laser power of 680 mW at the sample and an accumulation time of typically one hour. Potential laser-induced damage of the phytochrome samples could be ruled out, since comparison of RR spectra before and after a series of measurements did not reveal any changes. For the photoconversion, the protein sample was brought to a required temperature (e.g. to ca. 140, 240 and 300 K to obtain Lumi-F, Meta-F, and Pr, respectively) and illuminated with a 780 nm laser diode for 3 - 5 minutes. Illumination time varied to achieve possibly full conversion. After that, the sample was cooled again to 90 K for measurement. Residual contributions from the non-photoconverted state were removed by manually weighted spectra subtraction using the OPUS software (Bruker).

IR spectroscopic measurements were carried out using a Bruker Tensor 27 FTIR spectrometer in the transmission mode. Measurement temperatures were chosen such that the target photoconversion products were stabilized. IR spectra were recorded either in the dark-adapted state or under continuous illumination with a 780 nm LED array. The difference spectra were obtained by 1:1 subtraction of the initial state spectrum from the illuminated state spectrum.

## 1.3. Protein X-ray crystallography

*Crystallization and crystal structure determination.* The Agp2-PCM variant Y205oCNF was methylated and crystals were grown according to our previously published conditions.[2] To improve the quality of the crystals, a seeding procedure was introduced in an analogue crystallization setup. The highest resolution

diffraction data of Y205oCNF was collected at the ESRF synchrotron (Grenoble, France) at beamline ID23-1[3] using a Dectris Eiger X 16M detector at the wavelength of 0.8856 Å. All images were indexed, integrated, and scaled using the XDS program package[3,4] and the CCP4[5] programs SCALA[6] and AIMLESS.[6] Crystals of the Y205oCNF variant belong to the orthorhombic space group $P2_12_12_1$ (cell parameters for Y205oCNF: a = 74.4 Å, b = 93.5 Å, c = 173.8 Å, α = β = γ = 90°). Table S2 summarizes the statistics for the crystallographic data collection and structural refinement. The structure determination was performed as described previously.[2] Here, wild-type (WT) Agp2-PCM (PDB ID 6G1Y) was used as the initial search model for initial phases obtained with PHASER[7] by molecular replacement (rotation, translation, rigid-body fitting). Simulated annealing with the resulting model was performed using a slow-cooling protocol and maximum likelihood target function, energy minimization, and B-factor refinement by the program PHENIX.[8] The crystallographic structure was modelled with TLS refinement,[9] using anisotropic temperature factors for all protein atoms. Restrained, individual B-factors were refined, and the crystal structure was finalized by the CCP4 program REFMAC5[10] and other programs of the CCP4 suite.[5] The agreement factors $R_{free}$ and $R_{cryst}$ of the final Agp2-PCM variant Y205oCNF model are 17.78 % and 20.56 %, respectively (Table S2). Manual rebuilding of the crystal structure model and electron density interpretation was performed after each refinement step using the program COOT.[5] All molecular graphic representations in this work were created using PyMOL.[11]

### 1.4. Computational methods

#### 1.4.1. Structural Modeling and Molecular Dynamics Simulation Protocol

Structural models of the Agp2 variants—W440pCNF and F463oCNF—were constructed based on the crystal structure of the wild-type Agp2-PCM in the Pfr state (PDB ID: 6G1Y). Missing regions in the amino acid sequence were modeled using SWISS-MODEL through 3D homology modeling,[12] ensuring accurate alignment with the Agp2 sequence. Specific mutations were introduced by replacing the native residues at positions 440 and 463 with the corresponding Stark reporter groups pCNF and oCNF, respectively. For the Y205oCNF variant, calculations were based on the crystal structure resolved in this work, which was used as the starting coordinate set. All three structural models were protonated using the Karlsberg2+ code.[13] Special care was taken in assigning the protonation states of His248 and His278, which were modeled as charge-neutral with a proton located at the ε-nitrogen. Additionally, the propionic acid group C (propC) of the biliverdin (BV) chromophore was manually protonated in accordance with prior spectroscopic data. Following a previously published protocol,[1] each model was solvated in a cubic box of TIP3P water molecules,[14] and neutralized by adding sodium and chloride ions. Energy minimization was performed using the conjugate gradient algorithm over 50,000 steps, with gradually decreasing positional restraints on heavy atoms until a final force constant of 0.10 kcal/mol/Å² was reached. The systems were then gradually heated to 300 K in five steps of 40 ps each, using Langevin dynamics for temperature control with a time

step of 2 fs.[15] During equilibration, positional restraints on all heavy atoms were progressively relaxed over 80,000 steps (160 ps). Finally, a 50-ns production run was carried out under periodic boundary conditions in the NPT ensemble at a pressure of 1.01325 bar, regulated using the Langevin piston barostat with a piston period of 50 ps and a damping constant of 25 ps.[16] Electrostatic and van der Waals interactions were truncated at 12 Å, and long-range electrostatics were computed using the particle mesh Ewald (PME) method. A pair list cutoff of 13.5 Å was used. From the 50-ns long trajectories only 1500 from the last 30 ns were used for further evaluation. All simulations employed the CHARMM36 force field for both the protein and solvent,[17] while parameters for the BV chromophore were adopted from previous work.[18] All simulations were carried out using NAMD version 2.10.[19]

### 1.4.2. QM/MM geometry optimization and estimation of vibrational transition dipole moments (TDMs)

From the 1500 frames generated during the MD simulation, 25 were randomly chosen as initial structures for geometry optimizations employing a hybrid quantum mechanics/molecular mechanics (QM/MM) method implented in the ChemShell package.[20] In these calculations, the QM region - treated at the B3LYP/6-31G* level of theory - included the BV chromophore, the side chains of Cys13 and Asp196, the pyrrole water and the pCNF/oCNF residue in the respective models. The surrounding protein environment, solvent, and ions were described using the CHARMM36 force field. During energy minimization, only atoms within a 20 Å radius of the N22 atom of the BV chromophore were allowed to move while the atomic position of the remaining atoms were kept fix. Geometry optimization in hybrid delocalized coordinates was performed using L-BFGS method,[21] implemented in the DL-FIND geometry optimization library.[22] QM/MM coupling was implemented using a charge-shifted scheme in combination with electrostatic embedding.[23] The QM/MM-optimized geometries were used as input for subsequent vibrational frequency calculations, which were carried out exclusively on the QM region at the B3LYP/6-31G* level of theory using GAUSSIAN16,[24] following established protocols. In addition to vibrational frequencies, these calculations also yielded the transition dipole moment (TDM) associated with the nitrile stretching mode of the reporter group. Force constant scaling, normal mode analysis, and corrections to the QM Hessian matrix were conducted using in-house software developed by our group.[25] A scaling factor of 0.953 was specifically applied to adjust the stretching frequencies of the nitrile group.[1]

### 1.4.3. Classical Electric Field strength computations

Following a previously published protocol,[1] the averaged electric field projected onto the nitrile bond of the vibrational Stark probes (oCNF or pCNF) were estimated for the three Agp2 variants using Coulombs law by summing and projecting the contributions of all point charges in the environment onto the nitrile bond vector. The 25 QM/MM geometry optimized structures were employed for these calculations.

## 2. Structural data

### 2.1. Crystal structure of Y205oCNF

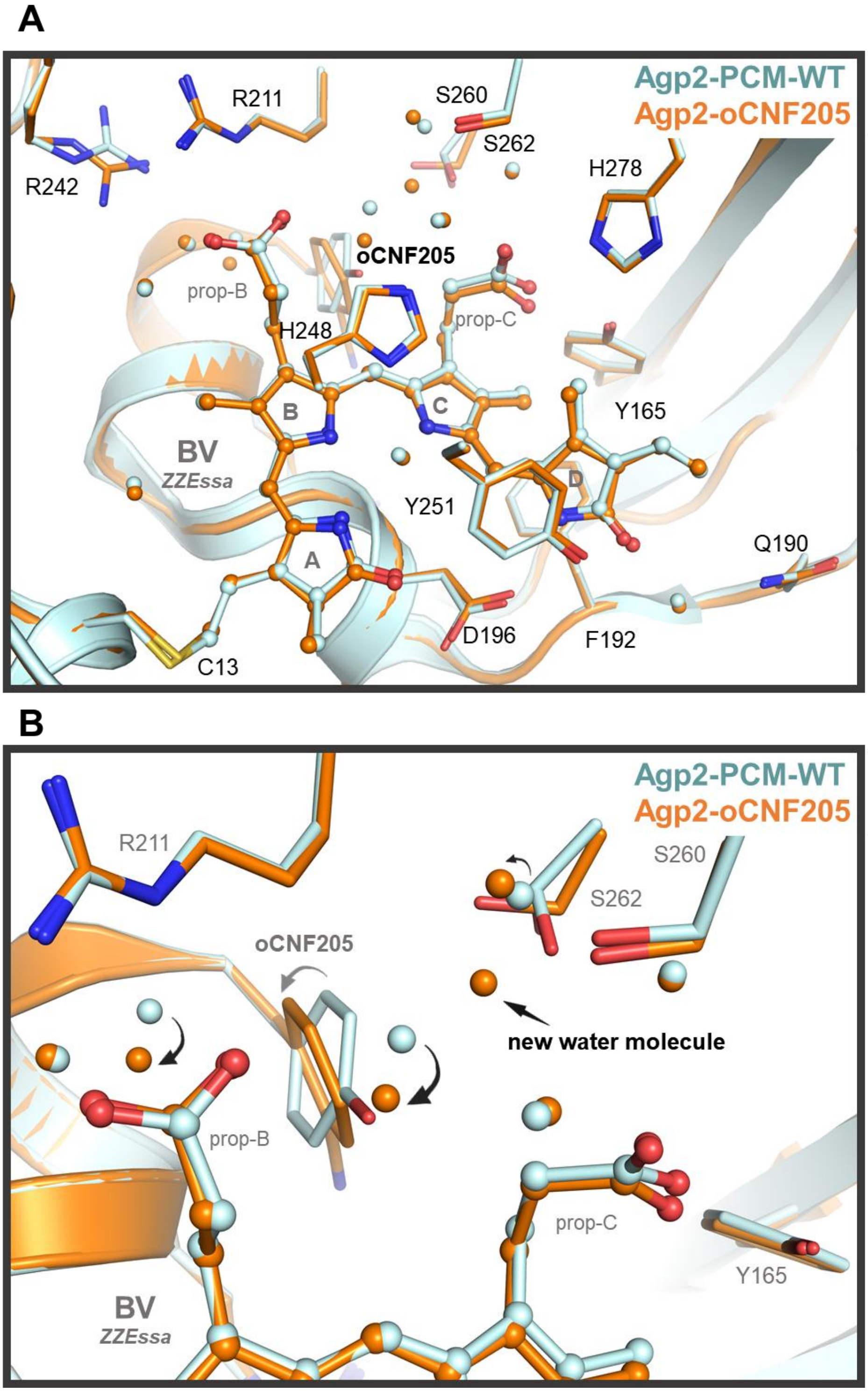


**Figure S1.** Crystal structure of the Pfr state of the Agp2-PCM variant Y205oCNF. (A) Superposition of the chromophore binding pockets of the WT Agp2-PCM (cyan) and the Y205oCNF variant (orange) crystal structures in their Pfr states. (B) The slight rotation of oCNF205 side chain causes a change of the hydrogen network around the amino acid leading to a insertion of a new water molecule. The protein backbone, chromophore, water molecules and selected residues are shown as cartoon, sticks/balls, spheres and sticks, respectively.

**Table S2.** Data collection and refinement statistics of the Stark label Agp2-PCM variant Y205oCNF

| | Y205oCNF[a]<br>(PDB ID 9RN8) |
|---|---|
| Data Collection | ESRF, ID23-1 |
| wavelength | λ = 0.8856 Å |
| Space group | $P2_12_12_1$ |
| Cell dimension | |
| *a*, *b*, *c* (Å) | 74.41, 93.54, 173.76 |
| α, β, γ (°) | 90.0, 90.0, 90.0 |
| Resolution (Å) | 41.22 - 1.791<br>(1.81 - 1.79)[b] |
| $\langle I/\sigma(I)\rangle$<br>*cc1/2* | 10.15 (1.30)<br>0.984 (0.724) |
| Completeness (%) | 99.88 (98.78) |
| Multiplicity | 6.9 (6.2) |
| Refinement | |
| No. reflections | 114259 |
| $R_{cryst}$ / $R_{free}$ (%) | 17.78/ 20.56 |
| No. atoms<br>Protein<br>Ligand/ion<br>Water | <br>7,675<br>172<br>975 |
| *B*-factors | |
| Protein<br>Ligand/ion<br>Water | 32.01<br>32.33<br>40.97 |
| RMSD[c] | |
| Bond lengths (Å) | 0.008 |
| Bond angles | 1.24 |
| Ramachandran plot[d] | |
| Favoured | 99.12 |
| Allowed<br>Outlier | 0.88<br>0.0 |

[a] One crystal was used; [b] highest resolution shell is shown in parenthesis; [c] RMSD, root mean square deviations; [d] Ramachandran plot calculated by MolProbity.[26]

## 2.2. Structural models of Y205oCNF, F463oCNF and W440pCNF

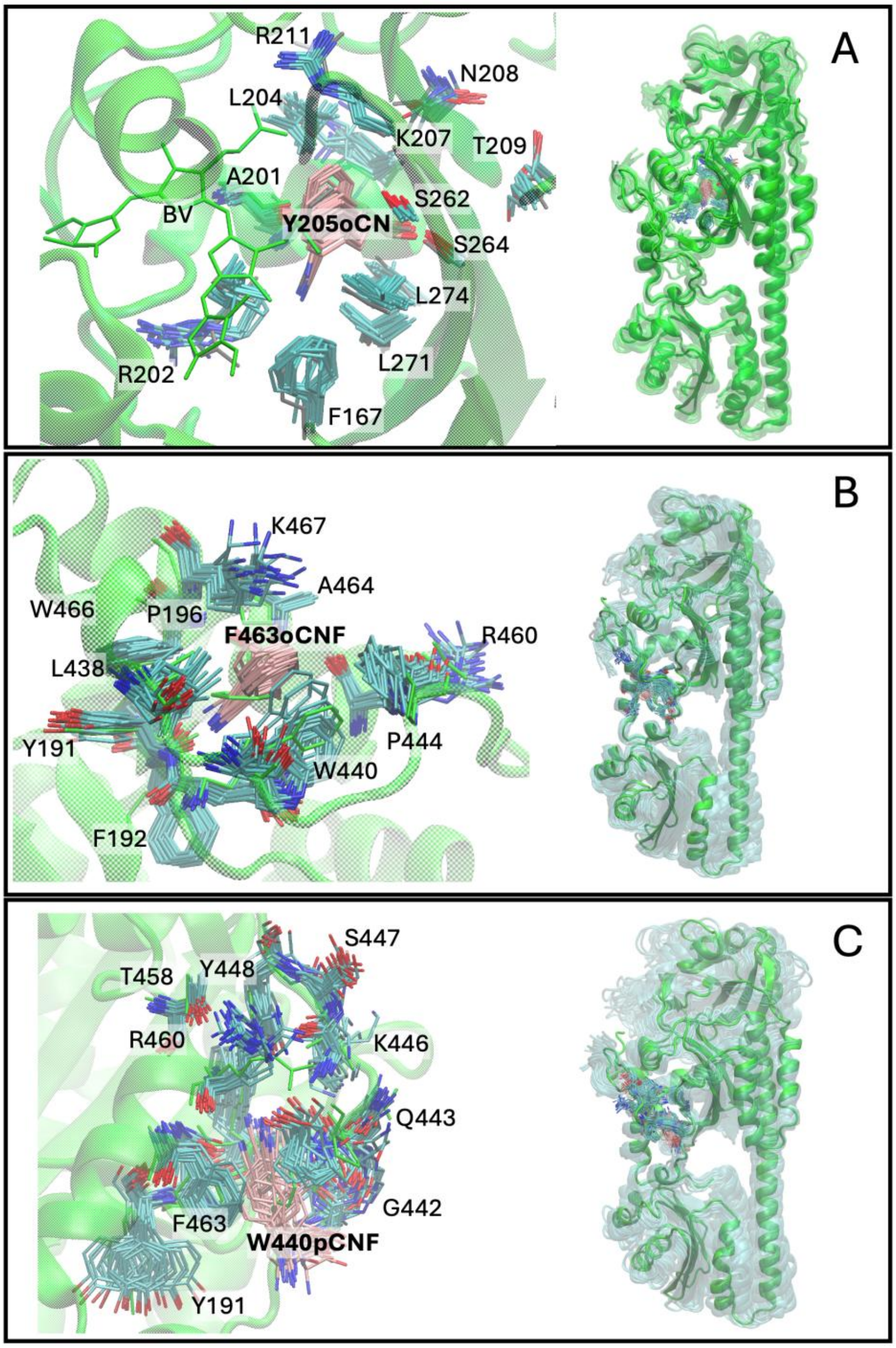


**Figure S2.** Conformational space of the Pfr states of the Agp2 variants (A) Y205oCNF, (B) F463oCNF and (C) W440pCNF as predicted from 25 QM/MM geometry-optimized snapshots extracted from MD simulations. The VSE reporter group is highlighted in pink, while surrounding key residues are shown in cyan. In (A) the crystal structure of the Y205oCNF variant is depicted as grey sticks, whereas in (B) and (C) the crystal structure of the wild type Agp2 is illustrated as green sticks. The full photoreceptor structure is depicted on the right, with a close-up view of the VSE reporter binding pocket shown on the left.

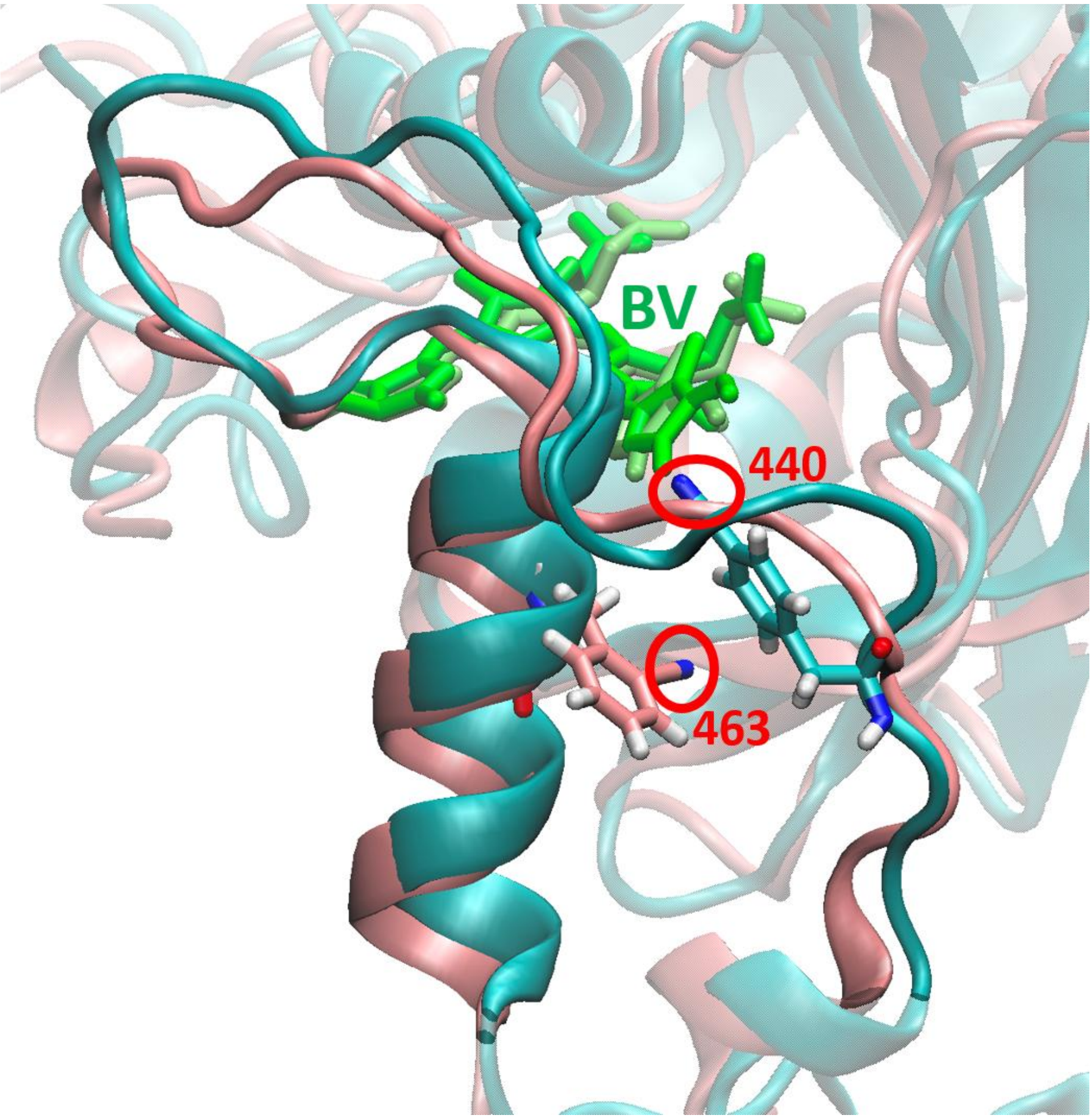


**Figure S3.** Orientation of VSE reporter groups (highlighted with red circles) in representative structural models of the Agp2 variants F463oCN (pink) and W440pCN (cyan). The BV chromophore is shown in green. The protein backbone is represented in cartoon format, while the oCN and pCN substitutions, along with the BV chromophore, are displayed in licorice representation. This image was generated using the VMD software.[27]

## 3. Spectroscopic data

### 3.1. UV-vis absorption spectroscopy

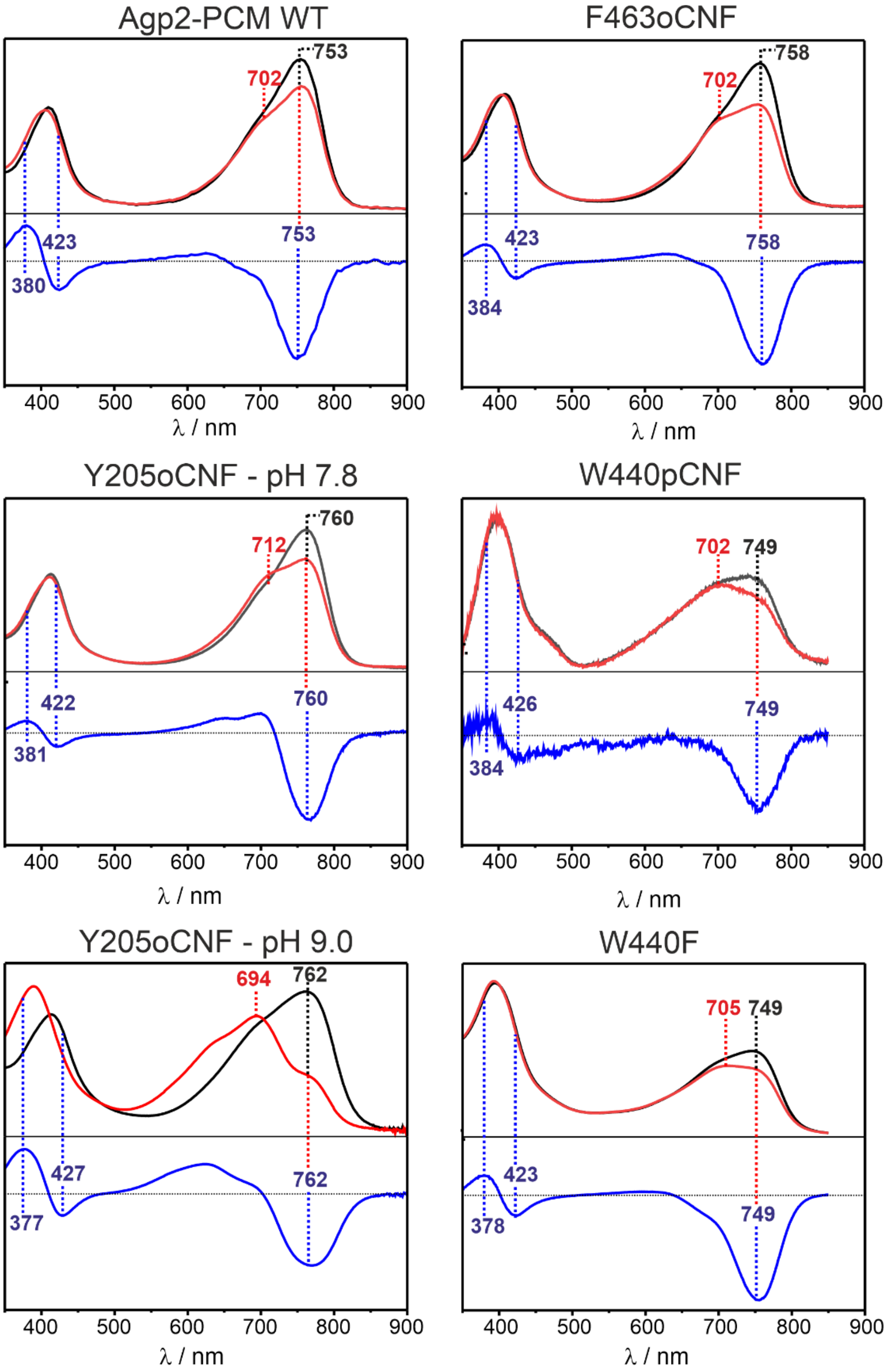


**Figure S4.** UV-vis absorption spectra of WT Agp2-PCM, Y205oCNF at pH 7.8 and 9.0, F463oCNF, W440pCNF, and W440F. The black and red traces represent the Pfr and Pr spectra, respectively. Blue traces denote the differences spectra. Note that the extent of photoconversion in WT Agp2-PCM is distinctly smaller as compared to the full-length protein, most likely due to the faster thermal back reaction from Pr to Pfr that occurs on the time scale of the measurement. A small photoconversion is also noted for the W440oCNF and W440F variants, although in this case a low photochemical quantum yield seems to be a more likely explanation. Interestingly, there is a substantial blueshift of the Pr absorption maxima upon increasing the pH from pH 7.8 to 9.0.

### 3.2. Resonance Raman spectroscopy

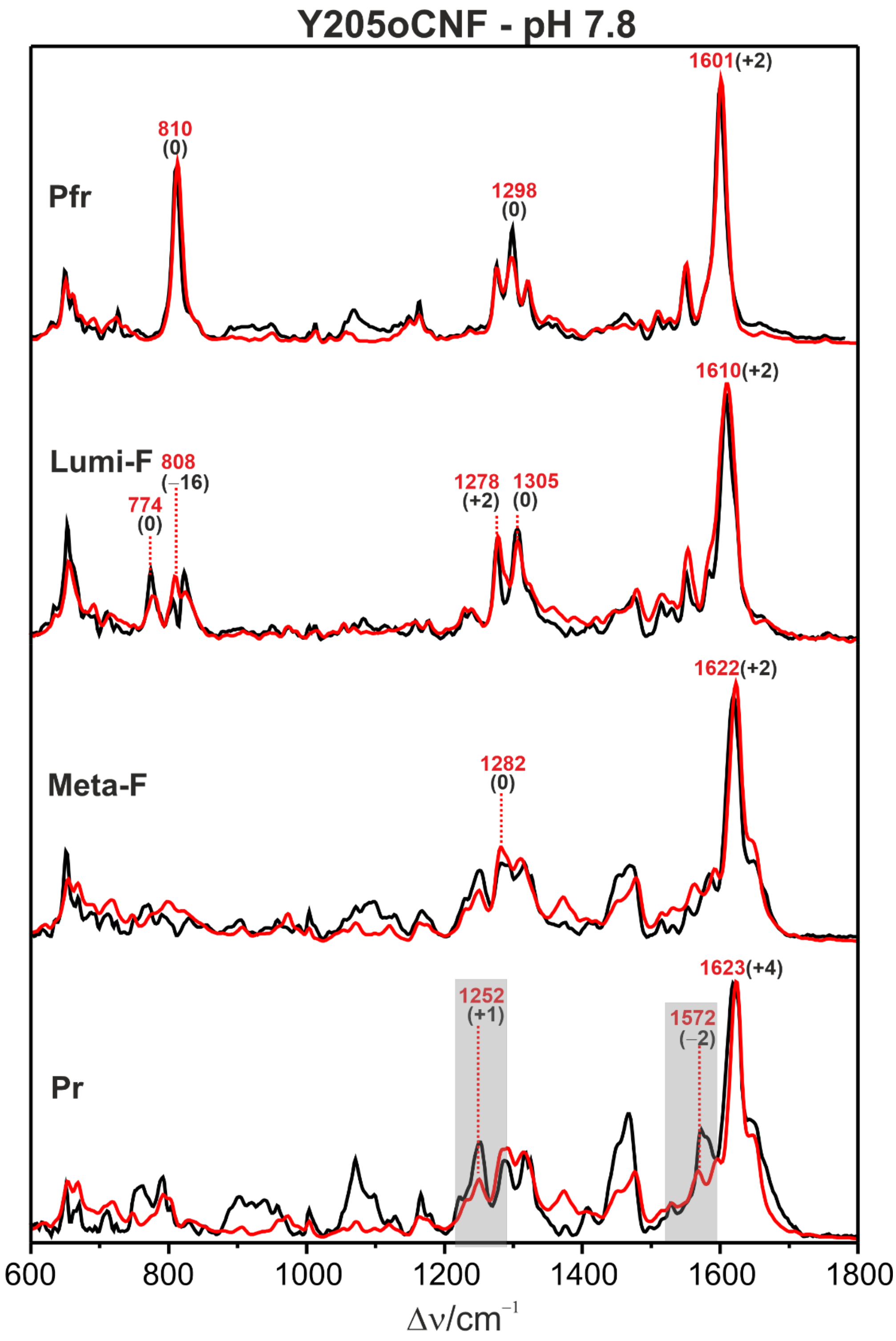


**Figure S5.** Overview RR spectra of Y205oCNF at pH 7.8 (red) compared with the WT Agp2-PCM (black). Red labels denote the peak maxima in the Y205oCNF spectrum whereas black numbers in parentheses indicate the frequency difference with respect to the corresponding peaks of the WT Agp2-PCM. The spectra were measured with 1064 nm excitation at 90 K. Photoinduced states were obtained by irradiation (750 nm) the sample at 140 K (Lumi-F), 240 K (Meta-F) and 300 K (Pr) prior to cooling to 90 K. Residual contributions of the unphotolyzed state Pfr were subtracted on the basis of the characteristic Pfr marker bands. The regions of the enol marker bands are highlighted in grey.

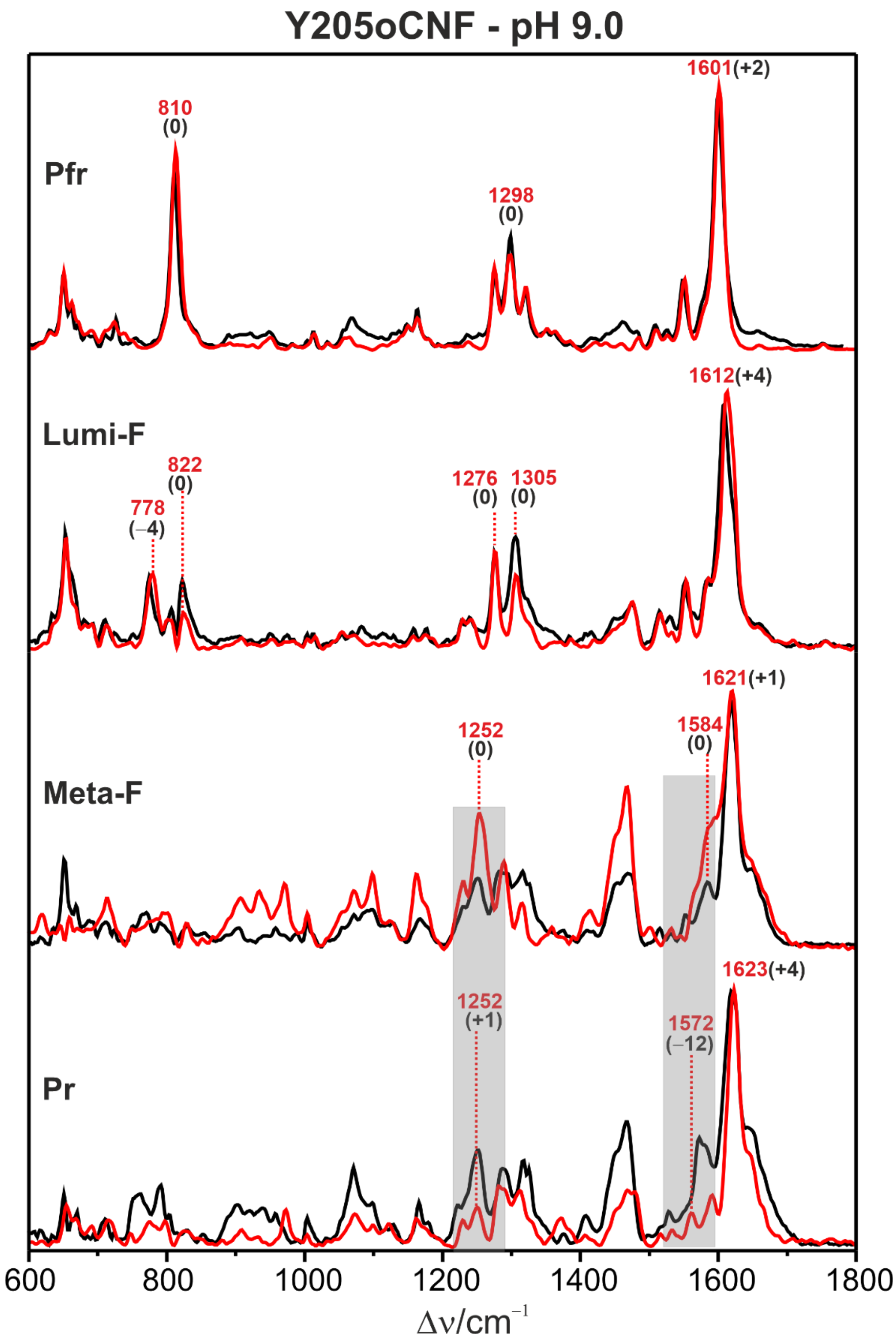


**Figure S6.** Overview RR spectra of Y205oCNF at pH 9.0 (red) compared with the WT Agp2-PCM (black). Red labels denote the peak maxima in the Y205oCNF spectrum whereas black numbers in parentheses indicate the frequency difference with respect to the corresponding peaks of the WT Agp2-PCM. The spectra were measured with 1064 nm excitation at 90 K. Photoinduced states were obtained by irradiation (750 nm) the sample at 140 K (Lumi-F), 240 K (Meta-F) and 300 K (Pr) prior to cooling to 90 K. Residual contributions of the unphotolyzed state Pfr were subtracted on the basis of the characteristic Pfr marker bands. The regions of the enol marker bands are highlighted in grey.

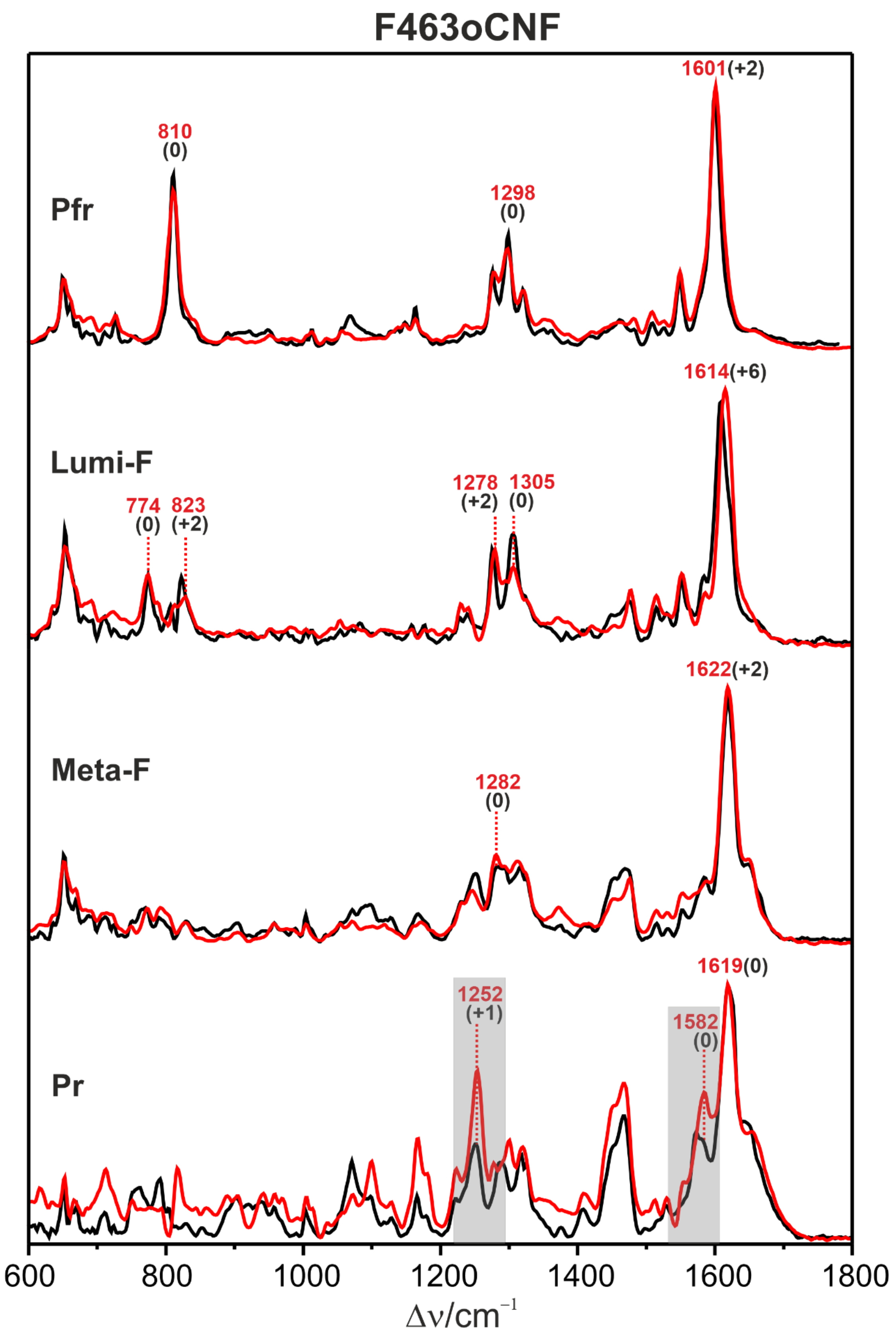


**Figure S7.** Overview RR spectra of F463oCNF at pH 7.8 (red) compared with the WT Agp2-PCM (black). Red labels denote the peak maxima in the Y205oCNF spectrum whereas black numbers in parentheses indicate the frequency difference with respect to the corresponding peaks of the WT Agp2-PCM. The spectra were measured with 1064 nm excitation at 90 K. Photoinduced states were obtained by irradiation (750 nm) the sample at 140 K (Lumi-F), 240 K (Meta-F) and 300 K (Pr) prior to cooling to 90 K. Residual contributions of the unphotolyzed state Pfr were subtracted on the basis of the characteristic Pfr marker bands. The regions of the enol marker bands are highlighted in grey.

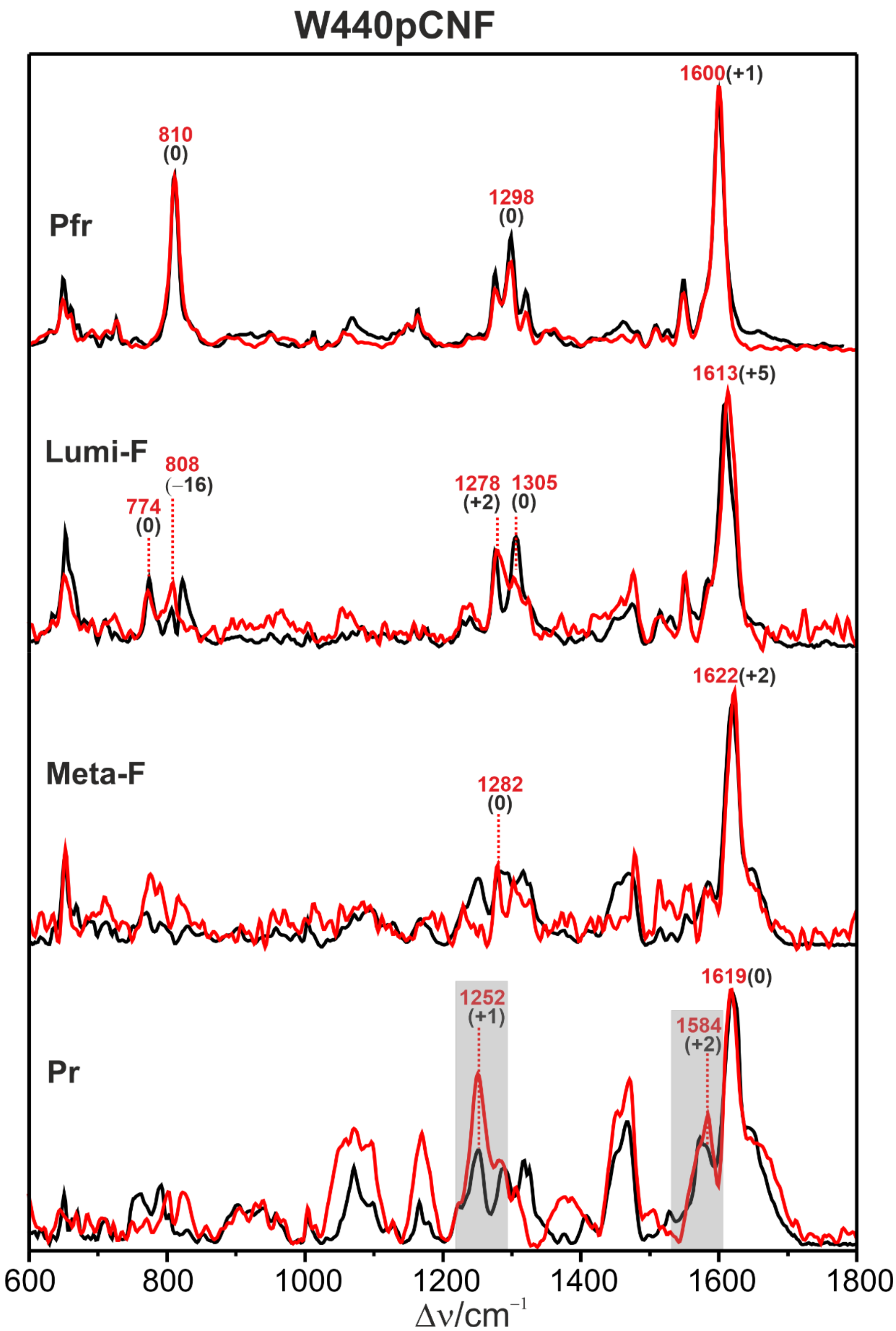


**Figure S8.** Overview RR spectra of W440pCNF at pH 7.8 (red) compared with the WT Agp2-PCM (black). Red labels denote the peak maxima in the Y205oCNF spectrum whereas black numbers in parentheses indicate the frequency difference with respect to the corresponding peaks of the WT Agp2-PCM. The spectra were measured with 1064 nm excitation at 90 K. Photoinduced states were obtained by irradiation (750 nm) the sample at 140 K (Lumi-F), 240 K (Meta-F) and 300 K (Pr) prior to cooling to 90 K. Residual contributions of the unphotolyzed state Pfr were subtracted on the basis of the characteristic Pfr marker bands. The regions of the enol marker bands are highlighted in grey.

## 3.3. IR spectroscopy

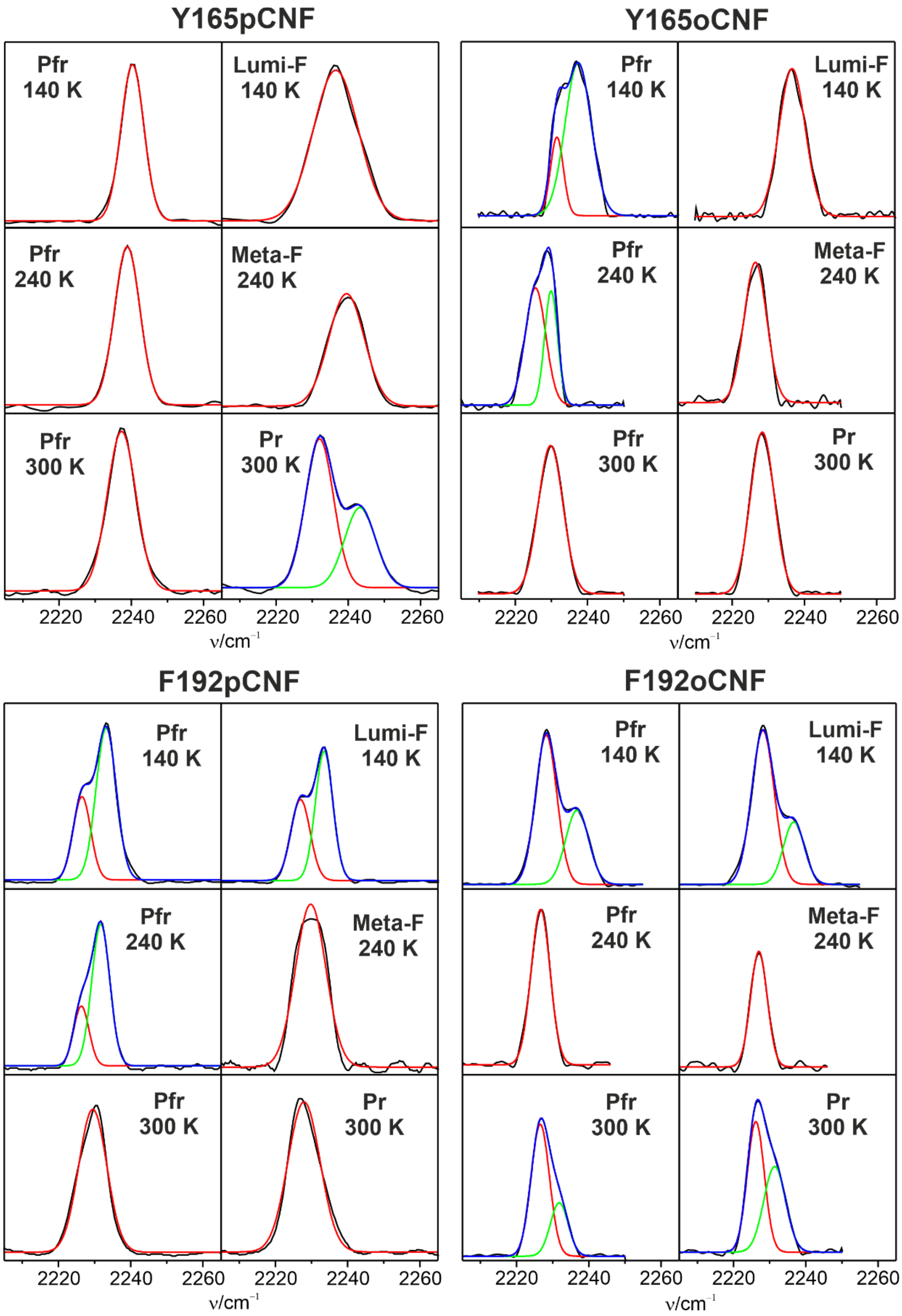


**Figure S9.** IR spectra in the nitrile stretching region of the Y165pCNF, Y165oCNF, F192pCNF, and F192oCNF variants. The experimental data as reported previously were re-analysed by subtracting the residual Pfr state contribution and subsequent band fitting. The experimental curves are given by the black lines whereas red and green traces represent the fitted Gaussian functions. In case of a two-Gaussian fit, the blue line refers to the sum of the individual Gaussians. The spectra of the photoproducts obtained at the indicated temperatures, were normalized to the conjugate spectra of the Pfr state.

## 4. Analysis of the nitrile stretching modes

We have revised the analytical methodology for translating experimental and calculated data of the nitrile stretching modes into electric fields. This revised methodology was also *a posteriori* applied to the variants studied in our previous work for the sake of consistency. However, it should be noted that essential results of the previous study[1] are not qualitatively affected.

### 4.1. Spectra subtraction

In the past, we always used the non-subtracted nitrile-stretching spectra tacitly assuming a 100 % conversion to the respective intermediate. This assumption is not justified. In the experiments, concentrations were comparable high as in the RR experiments (a few mg per mL), corresponding to an optical density in the visible (wavelength of the photoconversion light) of 10 or more. Hence, LED light (750 nm) used for photoconversion does not transmit through the sample. In RR spectroscopy using backscattering geometry, the penetration depth of the 1064 nm excitation line is larger such that the resultant RR spectra represent a mixture of photolyzed and unphotolyzed protein. This is the same for the IR spectra in the nitrile stretching region. In RR spectroscopy this problem can readily be overcome since subtraction of the residual Pfr contribution is straightforward due to the well-resolved and sharp bands of Pfr. In the case of the nitrile stretching modes, proper subtraction of the Pfr contribution is more difficult since the respective bands typically overlap. Thus, we used a subtraction procedure that was guided by (i) the subtraction factors $F_S$ in the RR experiments and (ii) the attempt to generate a symmetric bandshape of the difference band that could be simulated by a single Gaussian function. Nevertheless, there is a considerable uncertainty in choosing the correct subtraction factor particularly for strongly overlapping bands of the dark Pfr and the irradiated (photoinduced) state. However, this has only minor consequences for the final results. First, when frequencies are similar for both states, this is also true for the frequency of the difference band does not strongly depend on $F_S$. Second, the relative intensity of the intermediate as the crucial spectral parameter is evaluated by dividing the intensity of the photoinduced state ($I_{pp}$) and by that of Pfr ($I_{Pfr}$), both diminished by the subtracted contribution of Pfr ($F_S \cdot I_{Pfr}$). One can easily estimate that the maximum error associated with the uncertainty in the typical range of $F_S$ is less than 10%.

Note that in the IR difference spectra, the extent of product formation was not a problem. Low photoproduct concentrations only affect the absolute intensities of the difference signals but we consider solely the relative intensities.

**Table S3.** Nitrile stretching frequencies of the various states of the nitrile-labelled variants[a].

| | Observed frequencies | | | | | Normalized frequencies at room temperature | | | |
|---|---|---|---|---|---|---|---|---|---|
| variant | Peak | Pfr | Lumi-F | Meta-F | Pr | Pfr | Lumi-F | Meta-F | Pr |
| T/K[b] | | 140 | 140 | 240 | 300 | 300 | 300 | 300 | 300 |
| Y165oCNF | P1 | 2231.5 (2231.5) | 2236.6 (2236.8)[b] | 2226.4 (2228.7)[b] | 2228.3 (2229.8) | 2229.8 | 2229.6 | 2225.8 | 2228.3 |
| | P2 | 2237.4 (2237.4) | - | - | - | | | | |
| F192oCNF | P1 | 2228.2 (2228.2) | 2228.1 (2228.2) | 2227.0 (2226.6) | 2226.2 (2226.6) | 2226.6 | 2226.5 | 2227.0 | 2226.2 |
| | P2 | 2236.8 (2236.8) | 2236.7 (2236.8) | - | 2231.4 (2231.8) | 2231.8 | 2231.7 | - | 2231.4 |
| Y165pCNF | P1 | 2240.3 (2240.3) | 2236.5 (2240.3) | 2239.5 (2238.9) | 2232.4 (2237.3) | 2237.3 | 2233.5 | 2237.9 | 2232.0 |
| | P2 | - | - | - | 2241.6 (2237.4) | - | - | - | 2243.3 |
| F192pCNF | P1 | 2226.5 (2226.5) | 2227.0 (2226.5) | 2229.8 (2231.7)[b] | 2227.9 (2229.5) | 2229.5 | 2225.4 | 2227.6 | 2227.9 |
| | P2 | 2233.2 (2233.2) | 2233.6 (2233.2) | | | | 2230.8 | | |
| Y205oCNF | P1 | 2224.9 (2224.9) | 2224.6 (2224.9) | 2223.8 (2224.1) | 2222.7 (2223.6) | 2223.6 | 2223.3 | 2223.7 | 2222.7 |
| Y205oCNF pH 9 | P1 | 2224.6 (2224.6) | 2224.5 (2224.6) | 2224.3 (2224.4) | 2227.1 (2224.0) | 2224.0 | 2223.9 | 2223.9 | 2227.1 |
| W440pCNF | P1 | 2236.4 (2236.4) | 2235.2 (2236.4) | 2230.0 (2229.9) | 2232.3 (2232.0) | 2232.0 | 2230.6 | 2232.1 | 2232.3 |
| | P2 | - | - | 2238.0 (2238.1) | | - | - | 2240.0 | - |
| F463oCNF | P1 | - | - | 2227.8 (2227.8) | 2226.6 (2229.3) | 2229.3 | 2229.4 | 2229.3 | 2226.6 |
| | P2 | 2234.2 (2234.2) | 2234.3 (2234.2) | 2237.3 (2236.3) | - | | | 2238.8 | |

[a] Frequencies are given in $cm^{-1}$; values in parenthesis refer to the spectra of Pfr measured at the same temperature.
[b] refers to envelope peak maximum

**Table S4.** Integral IR intensities and their normalized values of the nitrile stretching modes in the various states of the nitrile-labelled variants.[a]

| variant | peak | Measured integral intensities $I_{i,m}$ | | | | | | Normalized intensities $I_i$ | | | |
|---|---|---|---|---|---|---|---|---|---|---|---|
| | | Pfr 140 K | Lumi-F 140 K | Pfr 240 K | Meta-F 240 K | Pfr 300 K | Pr 300 K | Pfr 300 K | Lumi-F 300 K | Meta-F 300 K | Pr 300 K |
| Y165oCNF | total | 0.00391 | 0.0019 (0.5) | 0.00279 | 0.00189 (0.25) | 0.01093 | 0.00672 (0.4) | 10.93 | 10.62 | 9.87 | 11.20 |
| | P1 | 0.00391 | 0.0019 | 0.00279 | 0.00189 | 0.01093 | 0.00672 | 10.93 | 10.62 | 9.87 | 11.20 |
| F192oCNF | total | 0.01687 | 0.00824 (0.5) | 0.00624[a] | 0.0029[a] (0.25) | 0.01286 | 0.00762 (0.5) | 12.86 | 12.56 | 7.969[a] | 15.25 |
| | P1 | 0.01096 | 0.006 | - | - | 0.00922 | 0.00415 | 9.22 | 9.148 | 7.969 | 8.3 |
| | P2 | 0.00591 | 0.00224 | - | - | 0.00364 | 0.00347 | 3.64 | 3.415 | | 6.94 |
| Y165pCNF | total | 0.00469 | 0.00898 (0.4) | 0.00315 | 0.00200 (0.1) | 0.02661 | 0.02869 (0.3) | 2.661 | 8.4672 | 1.8638 | 4.0986 |
| | P1 | - | - | - | - | - | 0.01802 | | - | - | 2.5743 |
| | P2 | - | - | - | - | - | 0.01067 | | - | - | 1.5243 |
| F192pCNF | total | 0.02704 | 0.01953 (0.15) | 0.00894 | 0.00666 (0.3) | 0.01535 | 0.01485 (0.2) | 1.535 | 1.337 | 1.589 | 1.856 |
| | P1 | 0.00869 | 0.00821 | 0.00235 | 0.00666 | 0.01535 | 0.01485 | | 0.543 | | |
| | P2 | 0.01835 | 0.01152 | 0.00659 | | | | | 0.794 | | |
| Y205oCNF | total | 0.03131 | 0.0142 (0.5) | 0.02321 | 0.01124 (0.5) | 0.00936 | 0.00583 (0.4) | 9.360 | 8.490 | 9.066 | 9.717 |
| | P1 | 0.03116 | 0.00142 | 0.02321 | 0.01124 | 0.00936 | 0.00583 | 9.36 | 8.490 | 9.066 | 9.717 |
| Y205oCNF pH9 | total | 0.0170 | 0.00948 (0.5) | 0.02238 | 0.01901 (0.15) | 0.00675 | 0.0076 (0.5) | 6.75 | 7.53 | 6.745 | 15.20 |
| | P1 | 0.0170 | 0.00948 | 0.02238 | 0.01901 | 0.00675 | 0.0076 | 6.75 | 7.53 | 6.745 | 15.20 |
| W440pCNF | total | 0.00383 | 0.001845 (0.5) | 0.0900 | 0.0475 (0.5) | 0.04122 | 0.01984 (0.5) | 4.122 | 3.969 | 4.349 | 3.968 |
| | P1 | 0.00383 | 0.001845 | 0.0361 | 0.0134 | 0.0412 | 0.01984 | 4.122 | 4.034 | 1.227 | 3.968 |
| | P2 | - | - | 0.0539 | 0.0341 | - | - | | | 3.122 | |
| F463oCNF | total | 0.05704 | 0.02438 (0.5) | 0.1221 | 0.0579 (0.5) | 0.01977 | 0.01751 (0.05) | 1.977 | 1.693 | 1.904 | 1.843 |
| | P1 | 0.05704 | 0.02438 | 0.03406 | 0.01984 | 0.01977 | 0.01751 | 1.977 | 1.84 | 0.6525 | 1.843 |
| | P2 | - | - | 0.08808 | 0.03806 | | | | | 1.2518 | |

[a] Values in parentheses are the subtraction factors that indicate the amount of subtracted Pfr spectrum ($F_S \cdot I_{Pfr}$). For the further analysis, in which the intensity of the spectrum photoinduced state is related to the Pfr spectrum measured at the same temperature, the amplitude of the Pfr spectrum was reduced by the same factor $F_S$.

**Table S5.** Normalized intensities, frequencies, and transition dipole moments of the various states of the nitrile-labelled variants.[a]

| variant | $\lvert\vec{m}_0\rvert$ | A | | | Pfr | Lumi-F | Meta-F | Pr |
|---|---|---|---|---|---|---|---|---|
| Y165oCNF | 4.44 | −0.047 | band envelope | $I_{\mathrm{i,tot}}$ | 10.93 | 10.62 | 9.87 | 11.20 |
| | | | | $\lvert\vec{\mathrm{m}}_{\mathrm{i,tot}}\rvert$ | 7.767 | 7.657 | 7382 | 7.862 |
| | | | | ν1 | 2229.8 | 2229.6 | 2225.8 | 2228.3 |
| F192oCNF | 4.44 | −0.047 | band envelope | $I_{\mathrm{i,tot}}$ | 12.86 | 12.56 | 7.969 | 15.25 |
| | | | | $\lvert\vec{\mathrm{m}}_{\mathrm{i,tot}}\rvert$ | 6.196 | 6.117 | 4.877 | 6.745 |
| | | | | ν1 | - | - | 2227.0 | - |
| | | | peak P1 | $x_1$ | 0.72 | 0.69 | - | 0.55 |
| | | | | ν1 | 2226.6 | 2226.5 | - | 2226.2 |
| | | | | $I_{\mathrm{i,1}}$ | 9.22 | 9.148 | - | 8.300 |
| | | | | $\lvert\vec{\mathrm{m}}_{\mathrm{i,1}}\rvert$ | 6.182 | 6.291 | - | 6.711 |
| | | | peak P2 | $x_2$ | 0.28 | 0.31 | - | 0.45 |
| | | | | ν2 | 2231.8 | 2231.7 | - | 2231.4 |
| | | | | $I_{\mathrm{i,2}}$ | 3.640 | 3.415 | - | 6.640 |
| | | | | $\lvert\vec{\mathrm{m}}_{\mathrm{i,2}}\rvert$ | 6.229 | 5.735 | - | 6.785 |
| Y165pCNF | 7.04 | −0.047 | band envelope | $I_{\mathrm{i,tot}}$ | 2.641 | 8.467 | 1.864 | 4.094 |
| | | | | $\lvert\vec{\mathrm{m}}_{\mathrm{i,tot}}\rvert$ | 9.564 | 17.122 | 8.033 | 11.912 |
| | | | | ν1 | 2237.3 | 2233.5 | 2237.9 | - |
| | | | peak P1 | $x_1$ | - | - | - | 0.63 |
| | | | | ν1 | - | - | - | 2232.0 |
| | | | | $I_{\mathrm{i,1}}$ | - | - | - | 2.5743 |
| | | | | $\lvert\vec{\mathrm{m}}_{\mathrm{i,1}}\rvert$ | - | - | - | 11.894 |
| | | | peak P2 | $x_2$ | - | - | - | 0.37 |
| | | | | ν2 | - | - | - | 2243.3 |
| | | | | $I_{\mathrm{i,2}}$ | - | - | - | 1.5243 |
| | | | | $\lvert\vec{\mathrm{m}}_{\mathrm{i,2}}\rvert$ | - | - | - | 11.943 |
| F192pCNF | 7.04 | −0.047 | band envelope | $I_{\mathrm{i,tot}}$ | 1.535 | 1.337 | 1.589 | 1.856 |
| | | | | $\lvert\vec{\mathrm{m}}_{\mathrm{i,tot}}\rvert$ | 8.829 | 8.241 | 8.983 | 9.709 |
| | | | | ν1 | 2224.9 | - | 2227.6 | 2227.9 |
| | | | peak P1 | $x_1$ | - | 0.4 | - | - |
| | | | | ν1 | - | 2225.4 | - | - |
| | | | | $I_{\mathrm{i,1}}$ | - | 0.543 | - | - |
| | | | | $\lvert\vec{\mathrm{m}}_{\mathrm{i,1}}\rvert$ | - | 8.305 | - | - |
| | | | peak P2 | $x_2$ | - | 0.6 | - | - |
| | | | | ν2 | - | 2230.8 | - | - |
| | | | | $I_{\mathrm{i,2}}$ | - | 0.794 | - | - |
| | | | | $\lvert\vec{\mathrm{m}}_{\mathrm{i,2}}\rvert$ | - | 8.199 | - | - |
| Y205oCNF | 6.33 | −0.039 | band envelope | $I_{\mathrm{i,tot}}$ | 9.360 | 8.490 | 9.066 | 9.717 |
| | | | | $\lvert\vec{\mathrm{m}}_{\mathrm{i,tot}}\rvert$ | 7.513 | 7.155 | 7.394 | 7.695 |
| | | | | ν1 | 2223.6 | 2223.3 | 2223.7 | 2222.7 |
| Y205oCNF pH9 | 6.33 | −0.039 | band envelope | $I_{\mathrm{i,tot}}$ | 6.75 | 7.53 | 6.745 | 15.20 |
| | | | | $\lvert\vec{\mathrm{m}}_{\mathrm{i,tot}}\rvert$ | 7.513 | 7.934 | 7.510 | 11.274 |
| | | | | ν1 | 2224.0 | 2223.9 | 2223.9 | 2227.1 |
| W440pCNF | 5.95 | −0.073 | band envelope | $I_{\mathrm{i,tot}}$ | 4.122 | 4.034 | 4.349 | 3.968 |
| | | | | $\lvert\vec{\mathrm{m}}_{\mathrm{i,tot}}\rvert$ | 12.819 | 12.582 | 13.170 | 12.580 |
| | | | | ν1 | 2232.0 | 2230.6 | - | 2232.3 |

| | | | | | | | | |
|---|---|---|---|---|---|---|---|---|
| | | | peak P1 | $x_1$ | - | - | 0.28 | - |
| | | | | ν1 | - | - | 2232.1 | - |
| | | | | $I_1$ | - | - | 1.227 | - |
| | | | | $\lvert\vec{\mathrm{m}}_{\mathrm{i},1}\rvert$ | - | - | 13.220 | - |
| | | | peak P2 | $x_2$ | - | - | 0.72 | - |
| | | | | ν2 | - | - | 2240.0 | - |
| | | | | $I_{\mathrm{i},2}$ | - | - | 3.122 | - |
| | | | | $\lvert\vec{\mathrm{m}}_{\mathrm{i},2}\rvert$ | - | - | 13.151 | - |
| F463oCNF | 4.23 | −0.073 | band envelope | $I_{\mathrm{i,tot}}$ | 1.977 | 1.693 | 1.904 | 1.843 |
| | | | | $\lvert\vec{\mathrm{m}}_{\mathrm{i,tot}}\rvert$ | 9.770 | 9.033 | 9.513 | 9.426 |
| | | | | ν1 | 2229.3 | 2229.4 | - | 2226.6 |
| | | | peak P1 | $x_1$ | - | - | 0.46 | - |
| | | | | ν1 | - | - | 2229.3 | - |
| | | | | $I_{\mathrm{i},1}$ | - | - | 0.6525 | - |
| | | | | $\lvert\vec{\mathrm{m}}_{\mathrm{i},1}\rvert$ | - | - | 8.211 | - |
| | | | peak P2 | $x_2$ | - | - | 0.54 | - |
| | | | | ν2 | - | - | 2238.8 | - |
| | | | | $I_{\mathrm{i},2}$ | - | - | 1.218 | - |
| | | | | $\lvert\vec{\mathrm{m}}_{\mathrm{i},2}\rvert$ | - | - | 10.496 | - |

[a] Intensities $I_i$ are given in arbitrary units, frequencies ν in cm$^{-1}$, and transition dipole moments $|\vec{m}_i|$ and $|\vec{m}_0|$ in $(km \cdot Mol)^{1/2}$. The subscript “tot” refers to the total intensity or TDM of a state $i$, i.e. in case of multiple bands, to the sum of the sub-states 1 and 2. The total TDM of the Pfr state ($\lvert\vec{\mathrm{m}}_{\mathrm{i,tot}}\rvert$ with I = Pfr) was calculated by averaging over the QMMM Pfr models. All other TDMs were evaluated from the intensities as using Eq. S2. The constant *A* is given in $\sqrt{\frac{km}{mol}} \cdot cm/MV$.

**Table S6.** Halfwidths (in cm$^{-1}$) of the Gaussian bandshapes of the nitrile stretchings in Pfr and Pr at room temperature.

| variant | Pfr (room temperature) | | Pr (room temperature) | |
|---|---|---|---|---|
| Y165oCNF | 6.8 | | 6.6 | |
| F192oCNF | 5.1 | 4.9 | 4.7 | 6.0 |
| Y165pCNF | 8.0 | | 7.8 | |
| F192pCNF | 7.8 | | 8.9 | |
| Y205oCNF | 4.8 | | 5.1 | |
| Y205oCNF pH 9 | 5.2 | | 8.7 | |
| W440pCNF | 9.6 | | 9.5 | |
| F463oCNF | 8.2 | | 7.3 | |

### 4.2. Stark tuning rate

For a state $i$ in a fully hydrophobic environment, the frequency shift $\Delta \nu_i$, corresponding to the difference of the observed frequency minus the zero-field frequency, should yield quantitatively the same electric field $E_{F,i}$ according to

$$\text{(S1)} \quad E_{F,i} = \frac{\Delta \nu_i}{|\overrightarrow{\Delta\mu}|}$$

And using the relative intensity according to

$$\text{(S2)} \quad |\vec{m}_{i,tot}| = \sqrt{\frac{I_{i,tot}}{I_{Pfr,tot}}}\,|\vec{m}_{Pfr,tot}|$$

$$\text{(S3)} \quad E_{F,i} = \frac{|\vec{m}_i| - |\vec{m}_0|}{A}$$

However, the use of the Stark tuning rate of $|\overrightarrow{\Delta\mu}| = 0.268\ cm^{-1}/(\mathrm{MV/cm})$, derived from model calculations in solvents in our previous work,[1] yielded substantial discrepancies when the results of Eq. (S1) and (S3) for Y205oCNF (pH 7.8) were compared. In that variant, each state is free of any hydrogen-bonding (HB) interactions. Therefore, we have re-determined $|\overrightarrow{\Delta\mu}|$ using the states of Y205oCNF (pH 7.8) and further purely hydrophobic states of other variants and obtained $|\overrightarrow{\Delta\mu}| = 0.37\ cm^{-1}/(\mathrm{MV/cm})$. This value is also much closer to the theoretical value,[28] and therefore we have employed it in the re-analysis of the data also for the previous variants.

### 4.3. Calculated electric fields and transition dipole moments

The structural models of Pfr states were used to calculate the electric fields projected onto the nitrile bonds of the Stark label $E_{F,Pfr,tot}$ and its transition dipole moment (TDM) $|\vec{m}_{Pfr,tot}|$. The average values from calculations of 25 – 50 snapshots of the MD simulation and QM/MM calculations are listed in Tables S5 and S7. Both $E_{F,Pfr,tot}$ and $|\vec{m}_{Pfr,tot}|$ are related to each other according to Eq. (S3). Plotting the individual data pairs yields the TDM in the absence of an electric field $|\vec{m}_0|$ and the proportionality constant $A$ (Figure S10, Table S7). For the three variants studied in this work, both quantities are different from those determined for the variants at position Tyr165 and Phe192 (see Table S5). Presumably, these values depend on the specific environment of the label.

In the previous work we have calculated the total electric fields of the photoproducts $E_{F,i,tot}$ according to Eq. S4

$$\text{(S4)} \quad E_{F,i,tot} = \frac{|\vec{m}_0| - |\vec{m}_{i,tot}|}{|\vec{m}_0| - |\vec{m}_{Pfr,tot}|} E_{F,Pfr,tot}$$

The results are associated with the error in $E_{F,Pfr,tot}$ *and* the TDMs, calculated for and derived from the Pfr calculations. To reduce the overall error, we have now calculated the electric fields of all states directly via Eq. S3 such that it is associated with the error of only one computed quantity, i.e. the TDM. This approach also allowed for determining the deviation (error) of the calculated electric field for Pfr and that derived from the relative IR intensities. In fact, in this way the error was distinctly reduced compared to the previous approach. Hence, we have employed this procedure also in the re-analysis of the previous data (Table S7).

Using Eq. S2, we first determined the TDMs of the transition for all variants and all states $i$ $|\vec{m}_{i,tot}|$, taking the integral relative intensity of the band envelope $I_{i,tot}$ as input. The results for $|\vec{m}_{i,tot}|$ obtained in this way were then used to evaluate the corresponding total electric fields $E_{F,i,tot}$ on the basis of Eq. S3.

The states involving discrete substates $j$ (i.e., multiple band components) need special attention. Eqs. S2 and S3 refer to molar quantities, that are the total intensity and TDMs of the transition and the total electric field. The experimental determined intensities of the substates $I_{i,j}$ represent only a fraction of the mole $x_j$. Hence, for each band component (substate) Eq. S2 is modified to

$$\text{(S5)} \quad |\vec{m}_{i,j}| = \sqrt{\frac{\frac{I_{i,j}}{x_j}}{I_{Pfr,tot}}} |\vec{m}_{Pfr,tot}|$$

or

$$\text{(S6)} \quad |\vec{n}_{i,j}| = |\vec{m}_{i,j}| \cdot \sqrt{x_j} = \sqrt{\frac{I_{i,j}}{I_{Pfr,tot}}} |\vec{m}_{Pfr,tot}|$$

Similarly, the electric fields of the substates $E_{F,i,j}$ are evaluated as molar quantities and thus are related to the total electric field of a state via the mole fractions. For the two-substate systems as in this work Eq. S7 holds.

$$\text{(S7)} \quad E_{F,i,tot} = x_1 \cdot E_{F,i,1} + (1 - x_1) \cdot E_{F,i,2}$$

Combining Eqs. S6 and S7 with Eq. S3 yields

$$\text{(S8)} \quad E_{F,i,tot} = \left( \frac{x_1}{A} \cdot \left( \frac{|\vec{n}_{1,i}|}{\sqrt{x_1}} - |\vec{m}_0| \right) \right) + \left( \frac{1-x_1}{A} \cdot \left( \frac{|\vec{n}_{i,2}|}{\sqrt{1-x_1}} - |\vec{m}_0| \right) \right)$$

which allows determining $x_j$ for the substates and subsequently, via Eqs. 6 and 3 $|\vec{m}_{i,j}|$ and $E_{F,i,j}$, respectively. All values for $|\vec{m}_{i,j}|$ and $x_j$ are listed in Table S5, whereas the values for $E_{F,i,j}$ are given in Table 1 of the manuscript.

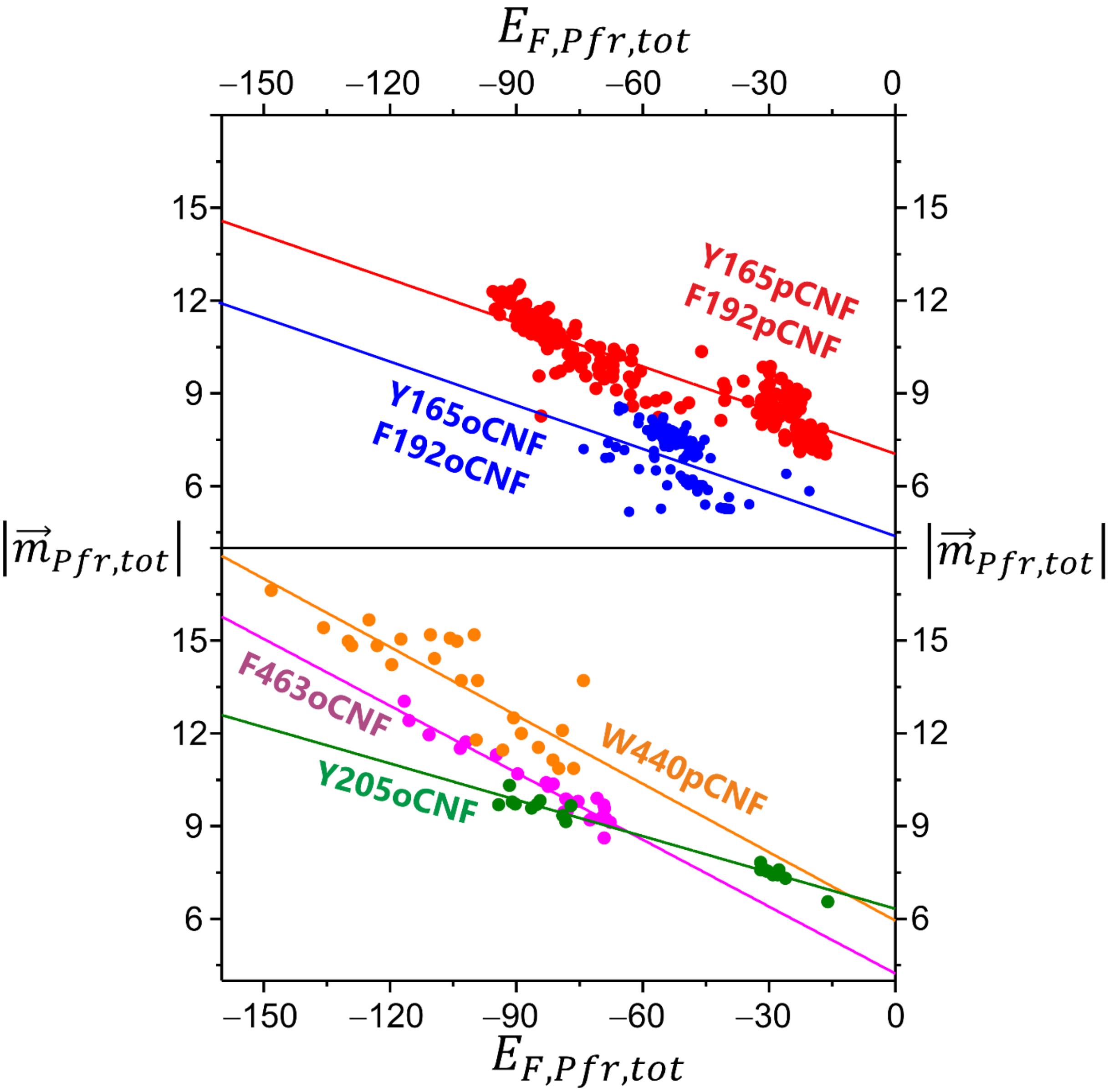


**Figure S10.** Plots of the TDMs vs. the electric fields for the Pfr states, calculated from 25 – 50 snapshots of the MD simulation. The upper panel displays the data for the variants reported previously whereas the variants investigated in the present study are shown in the lower panel. TDMs are given in $(km \cdot Mol)^{1/2}$ while electric fields in MV/cm.

**Table S7.** Calculated total electric fields for the Pfr states and the corresponding quantities for the intermediates using either the reference state Pfr (Eq. S4) or the transition dipole moment (Eq. S3)[a,b]

| | $E_{F,Pfr,tot}$ (MV/cm) | | $E_{F,Lumi,tot}$ (MV/cm) | | $E_{F,Meta,tot}$ (MV/cm) | | $E_{F,Pr,tot}$ (MV/cm) | |
|---|---|---|---|---|---|---|---|---|
| | Calc. av. | Eq. S3[b] | Eq. S4[a] | Eq. S3[b] | Eq. S4[a] | Eq. S3[b] | Eq. S4[a] | Eq. S3[b] |
| Y165oCNF | –53.41 | –70.79 | –51.64 | –68.45 | –47.22 | –62.59 | –54.94 | –72.82 |
| F192oCNF | –45.4 | –37.36 | –43.35 | –35.71 | –11.31 | –9.306 | –59.59 | –49.03 |
| Y165pCNF | –73.84 | –53.70 | –294.94 | –214.50 | –29.05 | –21.13 | –142.53 | –103.66 |
| F192pCNF | –28.76 | –38.06 | –19.31 | –25.56 | –31.25 | –41.35 | –42.91 | –56.79 |
| Y205oCNF | –30.15 | –30.33 | –21.47 | –21.16 | –27.11 | –27.28 | –33.76 | –33.97 |
| Y205oCNF pH9 | –30.15 | –30.33 | –40.89 | –41.14 | –30.08 | –30.27 | –126.01 | –126.77 |
| W440pCNF | –102.75 | –94.10 | –99.21 | –90.86 | –108.00 | –98.91 | –99.23 | –90.83 |
| F463oCNF | –71.38 | –75.89 | –61.89 | –65.80 | –68.95 | –71.43 | –66.95 | –71.18 |

**Table S8.** Summary of the values for $|\vec{m}_0|$ and *A* for all seven variants.

| Variante | $|\vec{m}_0|$ / $(km \cdot Mol)^{1/2}$ | $A$ / $\sqrt{\frac{km}{mol}} \cdot cm/MV$ |
|---|---|---|
| Y165oCNF | 4.44 | –0.047 |
| F192oCNF | 4.44 | –0.047 |
| Y165pCNF | 7.04 | –0.047 |
| F192pCNF | 7.04 | –0.047 |
| Y205oCNF | 6.33 | –0.039 |
| F463oCNF | 4.23 | –0.073 |
| W440pCNF | 5.95 | –0.073 |

**Table S9.** Error of the electric field calculations of Pfr

| | $E_{F,Pfr,tot}$ (MV/cm) | | Error with respect to 1st column | Error with respect to 2nd column |
|---|---|---|---|---|
| | Calc. av. | Eq. S3 | Dev. / % | Dev. / % |
| Y165oCNF | –53.41 | –70.79 | 32.54 | 24.55 |
| F192oCNF | –45.4 | –37.36 | 17.71 | 21.52 |
| Y165pCNF | –73.84 | –53.70 | 27.28 | 37.50 |
| F192pCNF | –28.76 | –38.06 | 32.34 | 24.44 |
| Y205oCNF | –30.15 | –30.33 | 0.60 | 0.59 |
| W440pCNF | –102.75 | –94.10 | 8.42 | 9.19 |
| F463oCNF | –71.38 | –75.89 | 6.32 | 5.94 |
| RMSD | | | 17.9±12.9 | 17.7±12.9 |

**Table S10.** Distances of the nitrile label from the carboxyl group of the propC side chain in Pfr obtained from MD simulations

| | Y165oCNF | F192oCNF | Y165pCNF | F192pCNF | Y205oCNF | W440pCNF | F463oCNF |
|---|---|---|---|---|---|---|---|
| Distance /Å | 5.9 | 4.5 | 10.4 | 6.7 | 9.8 | 14.9 | 14.9 |

## 4.4. Non-covalent and hydrogen-bonding-related electric fields

Separation of the non-covalent (subscript "non") and HB-related (subscript "HB") contributions to the electric field is based on the assumption that both components combine additively to yield the total electric field of a state or substate.[1]

(S9) $E_{F,i,tot} = E_{F,i,HB} + E_{F,i,non}$

This implies that the observed frequency shift $\Delta\nu_i$ (relative to the frequency in the absence of an electric field –2234.5 cm$^{-1}$) is the sum of the non-covalent and HB-related shifts

(S11) $\Delta\nu_i = \Delta\nu_{HB} + \Delta\nu_{non}$

Substituting $\Delta\nu_{non}$ by Eq. S1 and $\Delta\nu_{HB}$ by an analogous empirical expression, one obtains

(S12) $\Delta\nu_i = k \cdot E_{F,i,HB} + |\overrightarrow{\Delta\mu}| E_{F,i,non}$

and with Eq. S9

(S13) $E_{F,non,i} = \frac{\Delta\nu_i - k \cdot E_{F,i,tot}}{|\overrightarrow{\Delta\mu}| - k}$

with the constant $k$ equal to –0.2 (MV/cm)$^{-1}$cm$^{-1}$.

Eqs. S9 and S13 can then be used to approximate non-covalent and HB-linked electric fields. In the previous work,[1] we have applied this approach only to bands with frequencies of ≥ 2226 cm$^{-1}$, assuming that all bands with lower frequencies represent purely HB-free states. In this work we have discarded this somehow arbitrary assumption and applied this procedure to all bands.

In Figure S11, the HB-dependent electric field divided by the total electric field is plotted vs. the frequency shift, restricting the plot to shifts to negative values. One can readily see that for a HB-dependent field portion of 0.0 (MV/cm) the frequency shift is ca. –12 cm$^{-1}$, corresponding to an observed frequency of 2222.5 cm$^{-1}$, i.e., lower than originally assumed. Note that in three cases, the procedure yielded small positive values for the HB-dependent field, due to the inherent approximations of the approach. In these cases, the HB-dependent field was set to 0.0 (MV/cm). The results of the electric field analysis in terms of non-covalent and HB origin are listed in Table 1 of the manuscript.

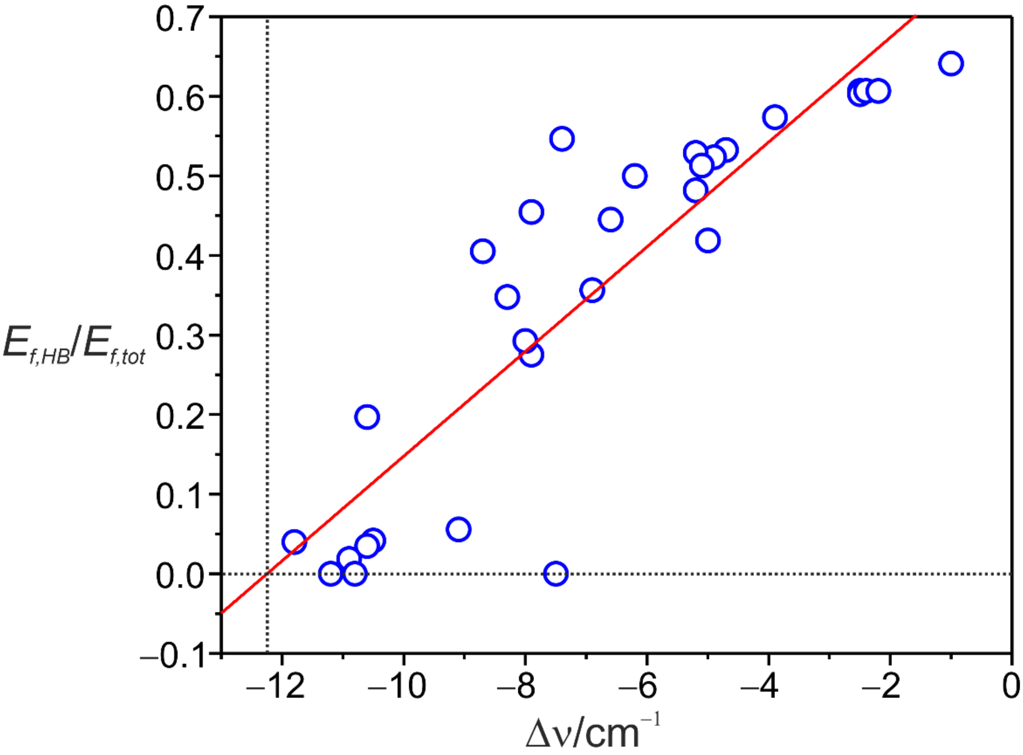


**Figure S11.** Portion of the HB-dependent field strength plotted against the observed frequency shift.

## 5. References


[1] A. D. Nguyen, N. Michael, L. Sauthof, J. von Sass, O. T. Hoang, A. Schmidt, M. La Greca, R. Schlesinger, N. Budisa, P. Scheerer, M. A. Mroginski, A. Kraskov, P. Hildebrandt, *J. Phys. Chem. B* **2024**, *128*, 11644–11657.

[2] A. Schmidt, L. Sauthof, M. Szczepek, M. F. Lopez, F. Velazquez Escobar, B. M. Qureshi, N. Michael, D. Buhrke, T. Stevens, D. Kwiatkowski, D. von Stetten, M. A. Mroginski, N. Krauß, T. Lamparter, P. Hildebrandt, P. Scheerer, *Nat. Commun.* **2018**, *9*, 1–13.

[3] D. Nurizzo, T. Mairs, M. Guijarro, V. Rey, J. Meyer, P. Fajardo, J. Chavanne, J. C. Biasci, S. McSweeney, E. Mitchell, *J. Synchrotron Radiat.* **2006**, *13*, 227–238.

[4] W. Kabsch, *Acta Crystallogr. Sect. D* **2010**, *66*, 125–132.

[5] Collaborative Computational Project, Number 4, *Acta Cryst. D* **1994**, *50*, 760–763.

[6] P. Evans, *Acta Crystallogr. Sect. D* **2006**, *62*, 72–82.

[7] A. J. McCoy, R. W. Grosse-Kunstleve, P. D. Adams, M. D. Winn, L. C. Storoni, R. J. Read, *J. Appl. Cryst* **2007**, *40*, 658–674.

[8] P. D. Adams, P. V Afonine, G. Bunkó, V. B. Chen, I. W. Davis, N. Echols, J. J. Headd, L.-W. Hung, G. J. Kapral, R. W. Grosse-Kunstleve, A. J. Mccoy, N. W. Moriarty, R. Oeffner, R. J. Read, D. C. Richardson, J. S. Richardson, T. C. Terwilliger, P. H. Zwart, *Acta Crystallogr. Sect. D* **2010**, *66*, 213–221.

[9] M. D. Winn, M. N. Isupov, G. N. Murshudov, *Acta Crystallogr. Sect. D Biol. Crystallogr.* **2001**, *57*, 122–133.

[10] G. N. Murshudov, A. A. Vagin, E. J. Dodson, *Acta Crystallogr. Sect. D Biol. Crystallogr.* **1997**, *53*, 240–255.

[11] The PyMOL Molecular Graphics, **2015**, Schrödinger, LLC.

[12] A. Waterhouse, M. Bertoni, S. Bienert, G. Studer, G. Tauriello, R. Gumienny, F. T. Heer, T. A. P. De Beer, C. Rempfer, L. Bordoli, R. Lepore, T. Schwede, *Nucleic Acids Res.* **2018**, *46*, W296–W303.

[13] B. Rabenstein, E. W. Knapp, *Biophys. J.* **2001**, *80*, 1141–1150.

[14] W. L. Jorgensen, *J. Am. Chem. Soc.* **1981**, *103*, 335–340.

[15] M. G. Paterlini, D. M. Ferguson, *Chem. Phys.* **1998**, *236*, 243–252.

[16] S. E. Feller, Y. Zhang, R. W. Pastor, B. R. Brooks, *J. Chem. Phys* **1995**, *103*, 4613–4620.

[17] A. D. MacKerell, N. Banavali, N. Foloppe, *Biopolymers* **2001**, *56*, 257–265.

[18] R. González, M. A. Mroginski, *J. Phys. Chem. B* **2019**, *123*, 9819–9830.

[19] J. C. Phillips, R. Braun, W. Wang, J. Gumbart, E. Tajkhorshid, E. Villa, C. Chipot, R. D. Skeel, L. Kalé, K. Schulten, *J. Comput. Chem.* **2005**, *26*, 1781–1802.

[20] S. Metz, J. Kästner, A. A. Sokol, T. W. Keal, P. Sherwood, *Wiley Interdiscip. Rev. Comput. Mol. Sci.* **2014**, *4*, 101–110.

[21] D. C. Liu, J. Nocedal, *Math. Program.* **1989**, *45*, 503–528.

[22] J. Kästner, J. M. Carr, T. W. Keal, W. Thiel, A. Wander, P. Sherwood, *J. Phys. Chem. A* **2009**, *113*, 11856–11865.

[23] H. W. Senn, W. Thiel, *Angew. Chemie Int. Ed.* **2009**, *48*, 1198–1229.

[24] M. J. Frisch, G. W. Trucks, H. B. Schlegel, G. E. Scuseria, M. A. Robb, J. R. Cheeseman, G. Scalmani, V. Barone, G. A. Petersson, H. Nakatsuji, X. Li, M. Caricato, A. V. Marenich, J. Bloino, B. G. Janesko, R. Gomperts, B. Mennucci, H. P. Hratchian, J. V. Ortiz, A. F. Izmaylov, J. L. Sonnenberg, D. Williams-Young, F. Ding, F. Lipparini, F. Egidi, J. Goings, B. Peng, A. Petrone, T. Henderson, D. Ranasinghe, V. G. Zakrzewski, J. Gao, N. Rega, G. Zheng, W. Liang, M. Hada, M. Ehara, K. Toyota, R. Fukuda, J. Hasegawa, M. Ishida, T. Nakajima, Y. Honda, O. Kitao, H. Nakai, T. Vreven, K. Throssell, J. A. J. Montgomery, J. E. Peralta, F. Ogliaro, M. J. Bearpark, J. J. Heyd, E. N. Brothers, K. N. Kudin, V. N. Staroverov, T. A. Keith, R. Kobayashi, J. Normand, K. Raghavachari, A. P. Rendell, J. C. Burant, S. S. Iyengar, J. Tomasi, M. Cossi, J. M. Millam, M. Klene, C. Adamo, R. Cammi, J. W. Ochterski, R. L. Martin, K. Morokuma, O. Farkas, J. B. Foresman, D. J. Fox, **2016**.

[25] M. A. Mroginski, F. Mark, W. Thiel, P. Hildebrandt, *Biophys. J.* **2007**, *93*, 1885–1894.

[26] I. W. Davis, A. Leaver-Fay, V. B. Chen, J. N. Block, G. J. Kapral, X. Wang, L. W. Murray, W. B. Arendall, J. Snoeyink, J. S. Richardson, D. C. Richardson, *Nucleic Acids Res.* **2007**, *35*, 375–383.

[27] W. Humphrey, A. Dalke, K. Schulten, *J. Mol. Graph.* **1996**, *14*, 33–38.

[28] S. D. Fried, S. G. Boxer, *Acc. Chem. Res.* **2015**, *48*, 998–1006.
